# Designing Nanostructured 3D Printed Materials by Controlling Macromolecular Architecture

**DOI:** 10.1002/anie.202206272

**Published:** 2022-07-18

**Authors:** Xiaobing Shi, Valentin A. Bobrin, Yin Yao, Jin Zhang, Nathaniel Corrigan, Cyrille Boyer

**Affiliations:** ^1^ Cluster for Advanced Macromolecular Design and Australian Centre for Nanomedicine School of Chemical Engineering University of New South Wales Sydney NSW 2052 Australia; ^2^ Electron Microscope Unit Mark Wainwright Analytical Centre University of New South Wales Sydney NSW 2052 Australia; ^3^ School of Mechanical and Manufacturing Engineering University of New South Wales Sydney NSW 2052 Australia

**Keywords:** 3D printing, nanostructure, RAFT polymerization, functional material

## Abstract

Nanostructured polymeric materials play important roles in many advanced applications, however, controlling the morphologies of polymeric thermosets remains a challenge. This work uses multi‐arm macroCTAs to mediate polymerization‐induced microphase separation (PIMS) and prepare nanostructured materials via photoinduced 3D printing. The characteristic length scale of microphase‐separated domains is determined by the macroCTA arm length, while nanoscale morphologies are controlled by the macroCTA architecture. Specifically, using 2‐ and 4‐ arm macroCTAs provides materials with different morphologies compared to analogous monofunctional linear macroCTAs at similar compositions. The mechanical properties of these nanostructured thermosets can also be tuned while maintaining the desired morphologies. Using multi‐arm macroCTAs can thus broaden the scope of accessible nanostructures for extended applications, including the fabrication of actuators and potential drug delivery devices.

## Graphical abstract

This work demonstrates photopolymerization induced microphase separation (PIMS) for 3D printed materials with nanostructures, which for the first time, are controlled by the structure of macromolecular chain transfer agent (MacroCTA). Importantly, phase‐inverted morphologies featuring precisely defined domain spacings are obtained by using multi‐arm macroCTA, which is not accessible with linear macroCTA.

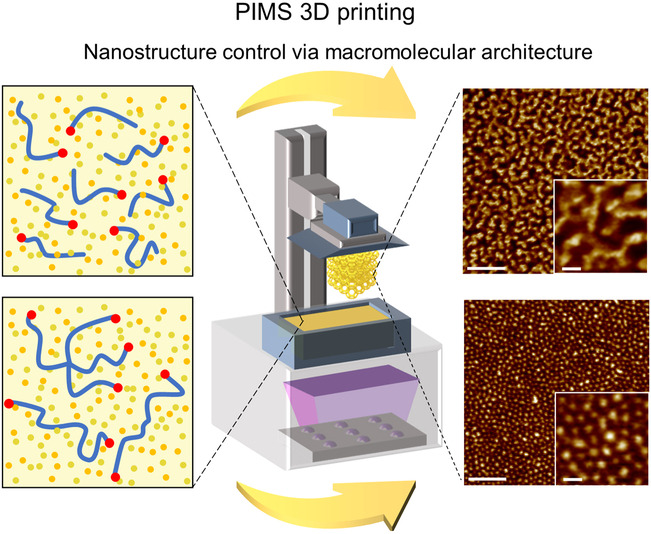

## Introduction

Nanostructured polymers featuring chemically distinct domains represent an increasingly useful category of materials. These materials are commonly prepared via self‐assembly of pre‐synthesized and well‐defined block copolymers in bulk using annealing processes,^[1]^ which provides materials with a wide range of ordered morphologies including spherical, cylindrical, gyroid, and lamellar structures. Correspondingly, these materials have broad applications in separation science, photonics, electronics, catalysis, and drug delivery.[^1^, ^2^] As an alternative to self‐assembly via annealing, polymerization‐induced phase separation (PIPS) processes provide a simple one‐step strategy to produce solid‐state nanostructured polymers from an initially homogeneous mixture of reagents. In these processes, the transition from homogeneous to phase‐separated states occurs as the polymerization proceeds.^[3]^ As a result of their nanostructure, materials made via PIPS have been used as superhydrophobic substrates,^[4]^ photonic crystals,^[5]^ and others.^[6]^ While PIPS processes are simple in practice, the scope of nanostructured morphologies is rather limited compared to block copolymer self‐assembly processes.[^2a^, ^7^] In this regard, the development of materials with more diverse morphologies via PIPS would be advantageous as it would expand the scope of potential applications for these materials.^[8]^

As a powerful subset of PIPS, polymerization‐induced microphase separation (PIMS) processes have been applied for producing mechanically robust thermosets with well‐defined nanostructures since the seminal work of Hillmyer and Seo in 2012.^[9]^ This technique relies on the chain extension of a macromolecular chain transfer agent (macroCTA) via reversible addition‐fragmentation chain transfer (RAFT) polymerization to provide block copolymers with thermodynamically incompatible block segments. These block copolymers undergo microphase separation before being kinetically arrested by in situ cross‐linking to form disordered microphase‐separated morphologies.[^9a^, ^10^] The properties of PIMS materials depends on their nanostructure,[^10^, ^11^] which can be influenced by many parameters, including macroCTA molecular weight,^[9]^ the type of comonomers^[11a]^ and cross‐linkers,^[9b]^ the presence of homopolymers,^[12]^ and reaction conditions.^[9b]^ By tuning these parameters, various nanostructured thermoset materials have been fabricated via PIMS for applications as porous materials[^9^, ^11a^, ^13^] and selective membranes,^[14]^ and in electrochemical[^10^, ^15^] and drug delivery systems,^[16]^ among others.^[11b]^

**Figure 1:**
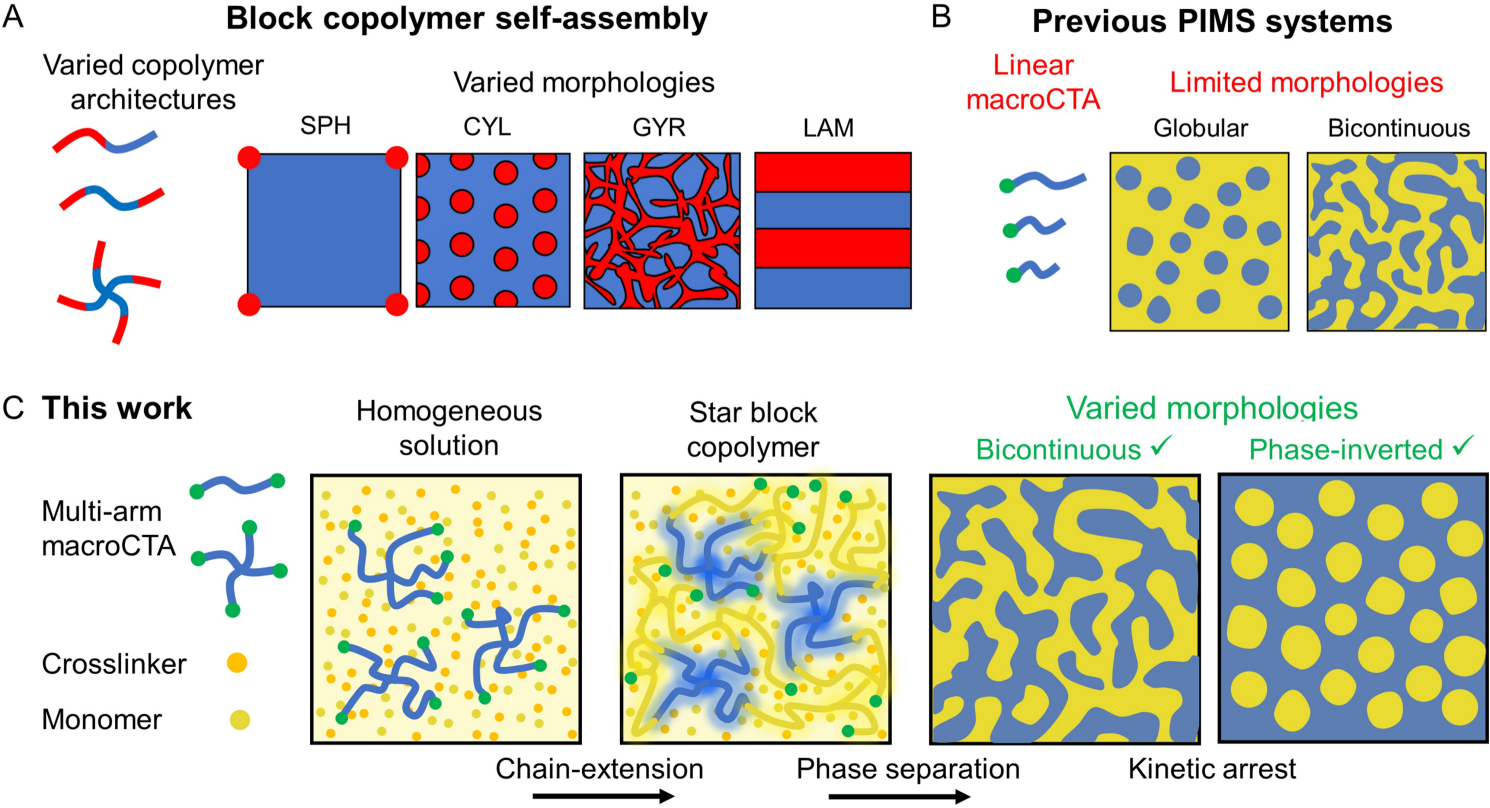
Strategies to produce nanostructured polymer materials. A) Block polymer self‐assembly for generation of various morphologies in non‐crosslinked systems. B) Previous polymerization‐induced microphase separation (PIMS) systems provide nanostructured materials, but with a limited range of morphologies. C) This work, using multi‐arm macroCTAs to mediate PIMS and provide access to nanostructured materials with phase‐inverted and bicontinuous morphologies via 3D printing.

Despite previous work demonstrating control over domain sizes and spacings for materials made via PIMS, the influence of the macroCTA architecture has not yet been investigated. This is somewhat surprising given that triblock (A−B−A) and star block (A−B)_n≥3_ copolymers are an important category of copolymers which have demonstrated interesting solid state self‐assembly behavior.^[17]^ For instance, Floudas and co‐workers demonstrated a slower ordering process for four‐arm star copolymers in comparison with linear diblock copolymers, which was attributed to the constrained chain mobility of star copolymers.^[18]^ In addition, Bates, Hawker, Bates and co‐workers recently evaluated the architectural effect on tetrahedrally close‐packed (TCP) sphere phases, finding significant morphological variation and a decrease in (χ*N*)_ODT_ upon increasing the number of arms in the block copolymers.^[19]^ Given the substantial influence of block copolymer architecture on their self‐assembly behavior (Figure 1A), we posited that the macroCTA architecture could also play an important role in the PIMS process and affect the resulting material nanostructure.

In parallel to these morphological developments, 3D printing has recently emerged as a viable production method for nanostructured materials, and one which allows increased design flexibility and more rapid material manufacture. For instance, Bates and co‐workers demonstrated that direct ink writing of bottlebrush polymers can be used to fabricate nanostructured elastomers, resulting in super‐soft materials with exceptional elastic behavior.^[20]^ Alternatively, Cavicchi and co‐workers,^[21]^ Levkin and co‐workers,^[22]^ and others,^[23]^ have demonstrated that PIPS can be applied to 3D printing to provide nanostructured materials with advanced functions such as triple shape memory,^[21]^ ultra‐hydrophobicity,^[22a]^ hierarchical porosity,^[22b]^ and electrical conductivity.^[23]^ Recently, our group developed a photoinduced PIMS process to generate nanostructured materials via digital light projection (DLP) 3D printing, which allowed the rapid fabrication of nanostructured materials with enhanced toughness.^[24]^ While these 3D printing processes provide geometrically complex materials in shorter times, there still remains a limitation in terms of final material morphologies (Figure 1B).

**Figure 2:**
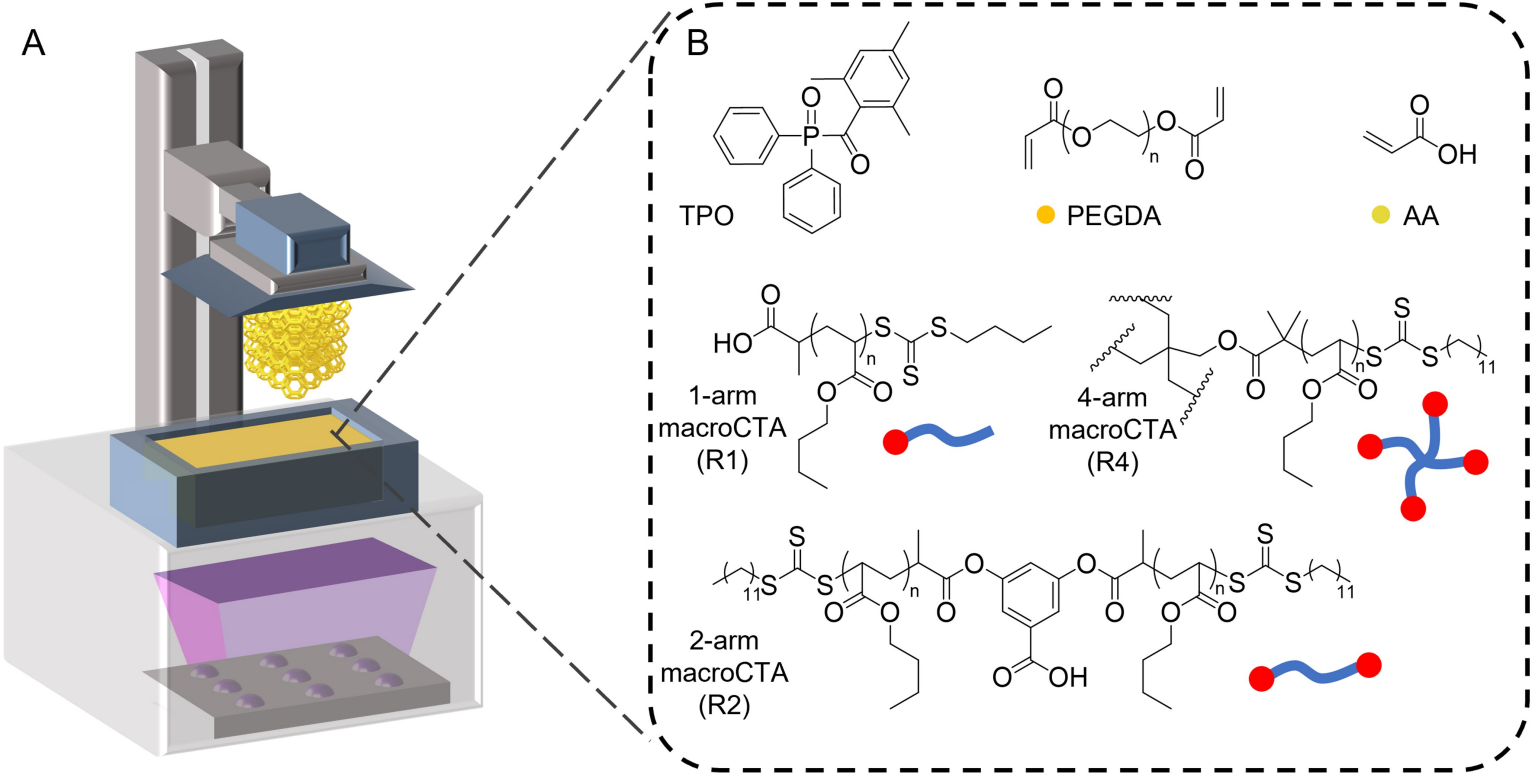
3D printing of PIMS materials mediated by multi‐arm macroCTAs. A) Schematic of DLP 3D printer used in this work. B) Chemical structures of resin components.

In this study, we investigated the effect of macroCTA architecture on the nanostructuration of materials produced via a photoinduced PIMS process. In particular, we used a DLP 3D printing process (Figure 2A) to induce PIMS and provide polymeric thermosets with controlled nanostructures. Different homogeneous resins were prepared by mixing pre‐synthesized macroCTAs with varied numbers of arms (1‐, 2‐, 4‐arms) and chain length per arm (*L*_ar*m*_), with a mixture of mono‐ and difunctional monomers and a photoinitiator. As evidenced by atomic force microscopy (AFM) and small‐angle X‐ray scattering (SAXS), the 3D printing process provided materials with controlled nanostructuration, where varying the macroCTA architecture, *L*_ar*m*_, and weight percentage (wt %) within resins led to 3D printed materials with nanoscale bicontinuous and phase‐inverted morphologies and tunable domain spacing (Figure 1C). Importantly, the phase‐inverted structures have not been previously observed in PIMS systems. Furthermore, the 3D printed PIMS materials demonstrated tunable mechanical properties and distinctive swelling behavior, which was dependent on the material nanostructuration. The enhanced solvent uptake of PIMS materials compared to non‐PIMS materials was successfully used to demonstrate swelling‐induced actuation and tailored release of model compounds.

## Results and Discussion

Three trithiocarbonate chain transfer agents, 2‐(*n*‐butylthiocarbonothioylthio) propanoic acid, 3,5‐bis(2‐dodecylthiocarbonothioylthio‐1‐oxopropoxy)benzoic acid and pentaerythritol tetrakis[2‐(dodecylthiocarbonothioylthio)‐2‐methylpropionate], were selected to synthesize 1‐arm (R1), 2‐arm (R2) and 4‐arm (R4) PBA macroCTAs, respectively, as shown in Figure 2B. As such, the chain extension of macroCTAs during photopolymerization was expected to produce (A−B)_*n*_ block copolymers, where n is 1, 2, and 4 for R1, R2 and R4, respectively. The different PBA macroCTAs were synthesized via thermal RAFT polymerization of *n*‐butyl acrylate (BA) using 2,2′‐azobisisobutyronitrile as thermal initiator (see Supporting Information for details). The number‐average molecular weight (*M*_n_) and dispersity (*Đ*), and the degree of polymerization (*X_n_*) of the synthesized macroCTAs were determined by size exclusion chromatography (SEC) (SI, Figure S1) and ^1^H NMR (SI, Figure S2). The values are reported in Table 1. The SEC and NMR results confirmed the synthesis of well‐defined macroCTAs with relatively close agreement between the experimental and theoretical values.

**Table 1:** Summary of PBA macroCTAs used in this study.

	MacroCTA	Conversion (%)^a^	*M*_n,theo._ (kg/mol)^b^	*X*_n,N*M*R_^c^	*M*_n,N*M*R_ (kg/mol)^d^	*M*_n,SEC_ (kg/mol)^e^	*Ð*
1 arm	R1‐90	94	12.3	94	12.3	9.6	1.11
	R1‐180	90	23.3	180	23.3	20.2	1.09
	R1‐360	90	46.4	360	46.4	38.9	1.19
2 arm	R2‐180	90	23.9	180	23.8	17.6	1.13
	R2‐360	85	44.4	340	44.3	32.7	1.10
4 arm	R4‐180	93	25.3	186	25.3	20.7	1.14
	R4‐360	90	47.7	360	47.6	35.3	1.17

Notes: [a] Monomer conversion was calculated by ^1^H NMR (SI, NMR, Figure S2). [b] *M*_n_,_theo_.=([BA]_0_/[RAFT agent]_0_)×conv. (BA)×MW(BA)+MW(RAFT agent). [c] The degree of polymerization (*X_n_*) of PBA‐CTAs was calculated by 1H NMR (SI, NMR, Figure S2). [d] *M*_n,N*M*R_=*X_n_* (PBA‐CTA)×MW(BA)+MW(RAFT agent). [e] SEC measurement was performed using DMAc (containing 0.03 % w/v LiBr and 0.05 % w/v 2,6‐dibutyl‐4‐methylphenol (BHT)) as eluent with polystyrene as calibration standards.

Photocurable resins were formulated using diphenyl(2,4,6‐trimethylbenzoyl)phosphine oxide (TPO) as photoinitiator, acrylic acid (AA) as monomer, poly(ethylene glycol) diacrylate (average *M*_n_=250 g/mol) as cross‐linker, and the synthesized PBA as macroCTA. For all resins, fixed molar ratios of [AA]/[PEGDA] at 4/1 and 0.5 wt % of TPO were used, while the macroCTA architecture, wt %, and *X_n_* were varied for comparison. The different resins were named using the following nomenclature, where the first two letters (R1, R2, and R4) represent the type of macroCTA, i.e., 1‐arm, 2‐arm, and 4‐arm, respectively, followed by the *X*_n_ and the wt % of macroCTA in the resin. For instance, R2‐180–28.2 refers to the resin containing 28.2 wt % of 2‐arm macroCTA in the resin with *X_n_* =180. The arm length was calculated by dividing the *X_n_* by the number of arms, e.g., R2‐180 has a *L*_ar*m*_=180/2=90. Upon violet light irradiation (405 nm), photoinduced RAFT polymerization allows controlled chain extension of macroCTAs with AA and PEGDA and generates block copolymers with distinct PBA and P(AA‐*stat*‐PEGDA) segments (Figure 1C). With growth of these copolymers, microphase separation between thermodynamically incompatible PBA and P(AA‐*stat*‐PEGDA) blocks occurs during the polymerization. At high monomer/cross‐linker conversion, the emergent nanostructure is kinetically arrested.

**Figure 3:**
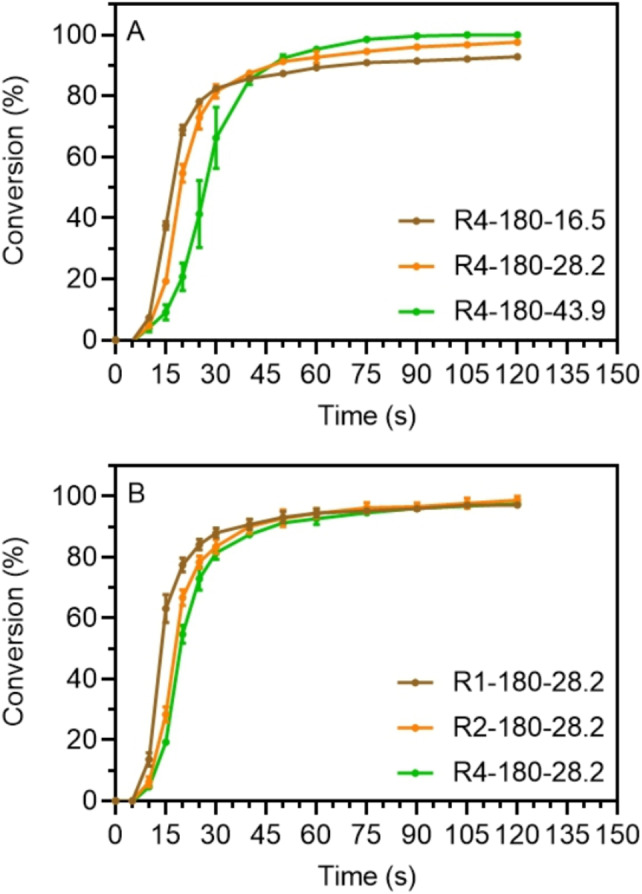
Polymerization kinetics of photocurable resins under violet irradiation (*λ*_max_=405 nm, *I*_0_=3.7 mW cm^−2^). A) Resins with different wt % of 4‐arm macroCTA (*X_n_* =180). B) Resins with a constant loading of 28.2 wt % of 1‐arm, 2‐arm and 4‐arm macroCTAs (*X_n_* =180).

Before implementation in 3D printing, the resin polymerization kinetics under violet light irradiation (3.7 mW/cm^2^, 405 nm) were investigated by attenuated total reflectance‐Fourier transform infrared (ATR‐FTIR) spectroscopy. Vinyl bond conversion was monitored by following the decrease in the absorption peak at ∼1620 cm^−1^ assigned to the stretching mode of the vinylic group (SI, Characterization). Figure 3A compares the polymerization kinetics of three resins with different R4‐180 wt %. Very high vinyl bond conversions (>80 %) were reached for these resins after 40 s of violet light irradiation with a negligible inhibition period (<5 s). Interestingly, resins with higher wt % of R4‐180 displayed a slower polymerization rate during the early stage of irradiation (<30 s), but showed higher final conversions compared to counterparts containing a lower R4‐180 wt %s. For example, the vinyl bond conversion reached ∼20 % after 20 s irradiation for R4‐180‐43.9, compared to ∼70 % for R4‐180‐16.5, while the conversion at plateau (120 s) of R4‐180‐43.9 was ∼100 %, compared to ∼93 % for the R4‐180‐16.5 system. A similar phenomenon was observed for resins with different R2‐180 wt %, where a slower polymerization rate was observed when using higher macroCTA wt % (SI, Figure S3A). Such retardation in systems containing higher macroCTA wt % was ascribed to the higher concentration of trithiocarbonate end groups, which competitively absorbs light at 405 nm, thus limiting the photoactivation of TPO and consequently reducing the concentration of propagating radicals.^[25]^ On the other hand, resins with lower macroCTA wt % contain a relatively higher concentration of cross‐linker, which leads to less mobile polymer networks that limit radical diffusion, thus limiting the conversion of vinyl groups.^[26]^

The influence of macroCTA structure was also studied by comparing the polymerization kinetics of resins with the same weight ratio of R1, R2, R4 at *X*_n_=180 or 360, respectively (Figure2B and SI, Figure S3B). It was found that using multi‐arm macroCTAs (R2, R4) resulted in a slight rate retardation in comparison with R1, but otherwise showed rapid polymerization. This was again attributed to a higher trithiocarbonate concentration and increased 405 nm light absorption, which was confirmed by UV/Vis measurement of three resins (SI, Figure S4). Overall, the polymerization kinetics study showed that all formulated resins can reach relatively high vinyl bond conversion (>80 %) in 40 s, indicating their potential to be applied for 3D printing.^[27]^

After establishing that the resins could be successfully cured in a relatively short time, we decided to print model objects, i.e., thin prisms with L×W×T=8×8×2 mm, using a commercially available 3D printer (Anycubic PhotonS, λ_max_=405 nm (violet light), *I*_0_=0.4 mW cm^−2^). 3D printing was performed under open‐air conditions using a layer thickness of 100 μm and curing time per layer of 180 s. All resins yielded well‐defined objects as shown in SI, Figure S5.

**Figure 4:**
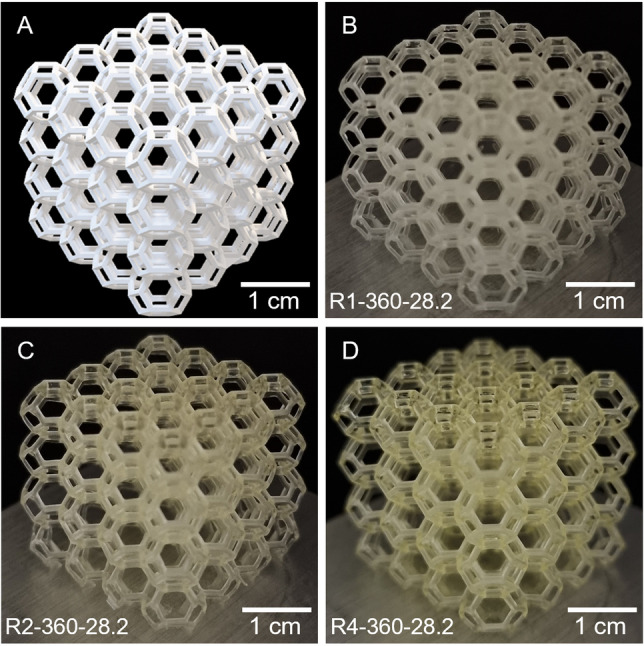
3D printing of complex lattice structures with sub‐millimeter features. A) Digital model of lattice structure. Lattice structures 3D printed using the resins: B) R1‐360‐28.2, C) R2‐360‐28. 2 and D) R4‐360‐28.2.

After successfully demonstrating 3D printing of the model objects, complex lattice structures were 3D printed using a higher resolution 3D printer (Photon MonoX, *λ*_max_=405 nm, 0.9 mW/cm^2^). Due to the higher light intensity in this 3D printer, the curing time per layer was fixed at 25 s while the layer thickness was maintained at 100 μm, providing a build speed of 1.44 cm/h. As shown in Figure 4B–D, the complex lattice structures were successfully prepared with high fidelity compared to the digital model, using the resins R1‐360‐28.2, R2‐360‐28.2, and R4‐360‐28.2. The tone of the 3D printed objects became darker when resins containing multi‐arm macroCTA were used, due to the increased concentration of trithiocarbonate groups in these resins. The measured strut thickness of the printed objects was 0.5–0.7 mm, demonstrating the ability of the developed PIMS resins to fabricate sub‐millimeter features. Given the high build rate and accurate reproduction of the digital models, the PIMS resins developed in this work appear suitable for the rapid production of geometrically complex objects.

**Figure 5:**
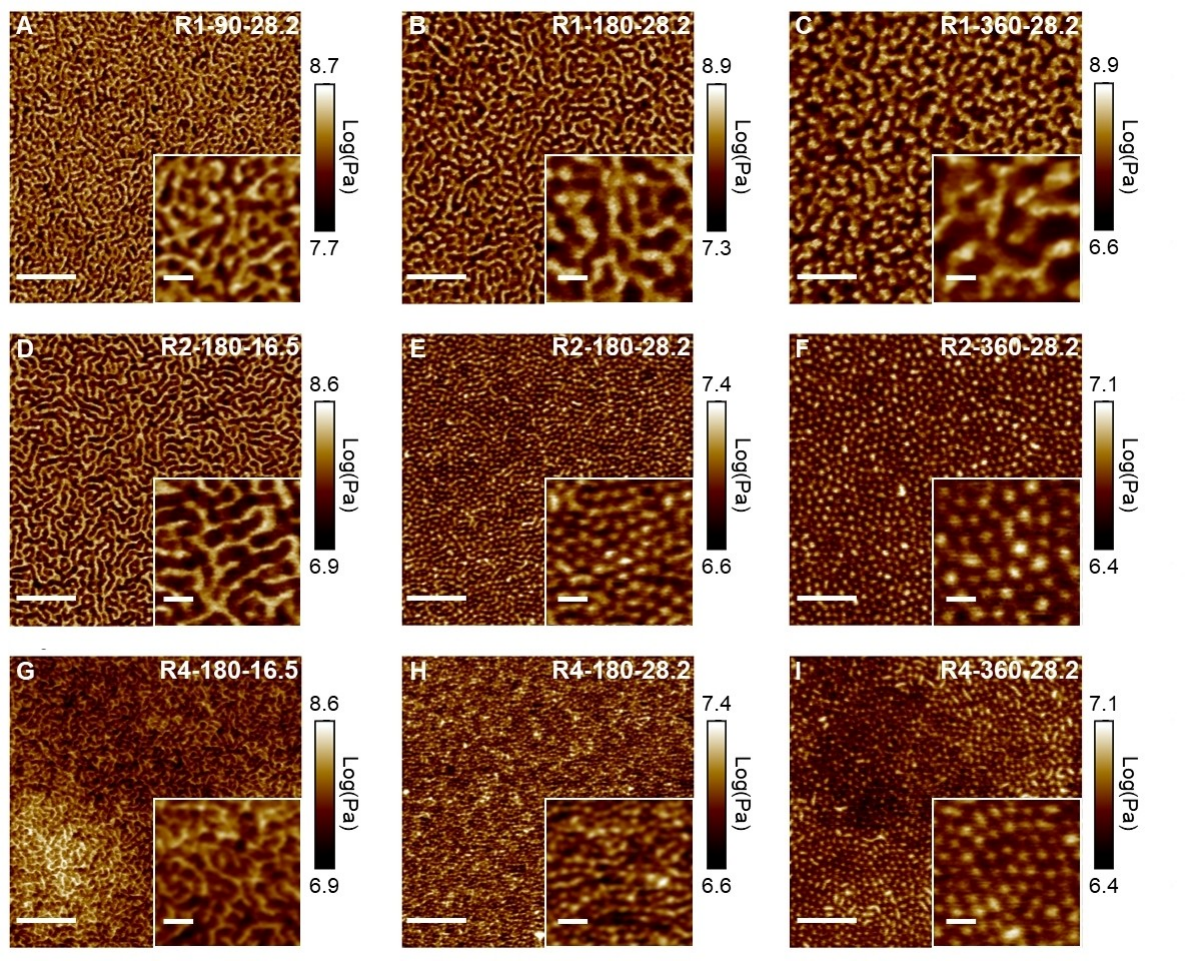
Surface morphologies of materials 3D printed using the resins: A) R1‐90‐28.2, B) R1‐180‐28.2, C) R1‐360‐28.2, D) R2‐180‐16.5, E) R2‐180‐28.2, F) R2‐360‐28.2, G) R4‐180‐16.5, H) R4‐180‐28.2, I) R4‐360‐28.2. The AFM images were obtained with PeakForce quantitative nanomechanics mode to acquire Log DMT modulus across scanned area. Dark area (low Log DMT modulus) refers to soft domain and light area (high Log DMT modulus) refers to hard domain. Inset: Magnified views of AFM images, Scale bar is 200 nm for main images and 40 nm for insets.

AFM measurements in PeakForce quantitative nanomechanics mode was used to investigate the nanostructure of 3D printed materials. It has been reported that soft PBA and hard PAA domains on 3D printed material surfaces can be distinguished due to elastic modulus differences.^[24]^ Detailed parameters applied in the AFM study can be found in the SI, Characterization section. As shown in Figure 5, AFM measurements of 3D printed rectangular prisms confirmed the presence of nanostructures for materials 3D printed using resins containing macroCTAs, whereas no phase‐separated morphology was observed for the control resins consisting of AA, PEGDA, BA, and multi‐arm RAFT agent (SI, Figure S6). This verified the critical role of macroCTAs in facilitating the PIMS process.^[24]^ Figure 5A–C show bicontinuous morphologies featuring soft PBA‐rich phase (dark area) and hard P(AA‐*stat*‐PEGDA)‐rich phase (light area) for materials 3D printed using R1‐based resins. These morphologies are in agreement with previously reported PIMS systems.[^9b^, ^12^] By increasing the *X*_n_ of R1 from 90 to 360, the measured PBA domain width (*D*_PBA_) increased from 13 to 24 nm, and the domain spacing (*d*_AF*M*_) increased from 22 to 50 nm (Table 2 and SI, Scheme S2). The influence of macroCTA *X_n_* on domain spacing was consistent with previously reported PIMS systems, which was ascribed to the increase in average block copolymer size when higher *X_n_* macroCTAs was used.[^11a^, ^16^]

**Table 2:** Morphologies and domain sizes observed by AFM for materials 3D printed using PIMS resins with linear monofunctional (R1) and multi‐arm (R2 or R4) macroCTAs.

	Formulation	*L_arm_*	Morphology^a^	*D*_PBA_ (nm)^*b*^	*d*_AFM_ (nm)^*b*^	*d*_SAXS_ (nm)^*c*^
1 arm	R1‐90‐28.2	90	Bicontinuous	13	22	21
	R1‐180‐28.2	180	Bicontinuous	15	33	31
	R1‐360‐28.2	360	Bicontinuous	24	50	56
2 arm	R2‐180‐16.5	90	Bicontinuous	13	23	22
	R2‐180‐28.2	90	Phase‐inverted	12	23	21
	R2‐180‐43.9	90	Phase‐inverted	^*d*^	^*d*^	19
	R2‐360‐28.2	180	Phase‐inverted	13	33	31
4 arm	R4‐180‐16.5	45	Bicontinuous	8	17	16
	R4‐180‐28.2	45	Bicontinuous/ Phase‐inverted	8	15	14
	R4‐180‐43.9	45	^*d*^	^*d*^	^*d*^	13
	R4‐360‐28.2	90	Phase‐inverted	11	25	21

Note: [a] Morphology of 3D printed materials determined by AFM. [b] PBA domain width (*D*_PBA_), domain spacing (*d*_AFM_) determined from AFM. [c] Domain spacing (*d*_SAXS_) determined from SAXS. [d] *D*_PBA_ and *d*_AFM_ values for R2‐180‐43.9 and R4‐180‐43.9 were not reported due to difficulty in precise measurement (SI, Figure S7).

Interestingly, samples 3D printed using R2‐180 resins displayed a distinctive morphology with reduced P(AA‐*stat*‐PEGDA) phase continuity when the wt % of R2 in resins was increased from 16.5 to 28.2 wt % (Figure 5D–E). Herein, we denote this morphology as phase‐inverted as the P(AA‐*stat*‐PEGDA)‐rich phase (light area) appeared to be surrounded by the PBA phase (dark area) in the AFM images (Figure 5D–E). This phase‐inverted morphology was maintained for materials 3D printed with R2‐180‐43.9 (SI, Figure S7A). A similar nanostructural transition from bicontinuous to phase‐inverted morphologies was observed for materials 3D printed materials using R4‐based resins when the macroCTA wt % in the starting resins was increased. However, using R4‐based resins resulted in materials featuring smaller *D*_PBA_ and *d*_AF*M*_ compared with those from R2‐based resins for similar *X_n_* and macroCTA wt %. For instance, the measured *D*_PBA_ and *d*_AF*M*_ for materials 3D printed with R2‐180‐16.5 were 13 and 23 nm, which reduced to 8 and 17 nm, respectively, when using R4‐180‐16.5 (Table 2). In addition, materials 3D printed using resins containing R2 and R4 at higher *X_n_* of around 360 as also displayed phase‐inverted morphologies (Figure 5F, I). Similarly, R2 and R4 based resins showed an increase in PBA domain width and domain spacing with increasing macroCTA *X_n_* (Table 2).

It is worth noting that due to the structural differences between the macroCTAs, the concentration of trithiocarbonate end groups in R2 and R4 resins are about twice and four times those of the R1 resins at the same macroCTA *X_n_* and wt %. In addition, the number of BA units in each arm (*L*_ar*m*_) of the R2 and R4 macroCTAs is half and a quarter of those in counterpart R1. macroCTAs at the same *X_n_*. In this sense, it is worthwhile comparing materials 3D printed using R1, R2, and R4 resins containing the same *L*_ar*m*_ and trithiocarbonate concentration. For example, the R1‐90‐28.2, R2‐180‐28.2, and R4‐360‐28.2 resins all have identical macroCTA arm lengths (*L*_ar*m*_=90) and weight ratio (28.2 wt %). As expected, these materials exhibited similar *D*_PBA_ and *d*_AF*M*_ values (Table 2), which supports the finding that the PBA domain width and domain spacing are mainly controlled by *L*_ar*m*_.

Although samples 3D printed using resins with the same *L*_ar*m*_ and macroCTA wt % showed similar domain spacings, their morphologies as observed in AFM were different. Indeed, only R1‐90‐28.2 gave a disordered bicontinuous morphology, while R2‐180‐28.2 and R4‐360‐28.2 showed phase‐inverted morphologies. Additionally, materials 3D printed using R2‐360‐28.2 showed a phase‐inverted morphology, while R1‐180‐28.2 featured a disordered bicontinuous morphology, despite their similar *L*_ar*m*_ values of 180. The R2‐360‐28.2 and R1‐180‐28.2 materials also displayed similar *D*_PBA_ and *d*_AF*M*_ values (Table 2). Therefore, when *L*_ar*m*_ is the same, the macroCTA architecture can provide an additional parameter to control the morphology, without affecting the domain spacing and PBA domain width.

**Figure 6:**
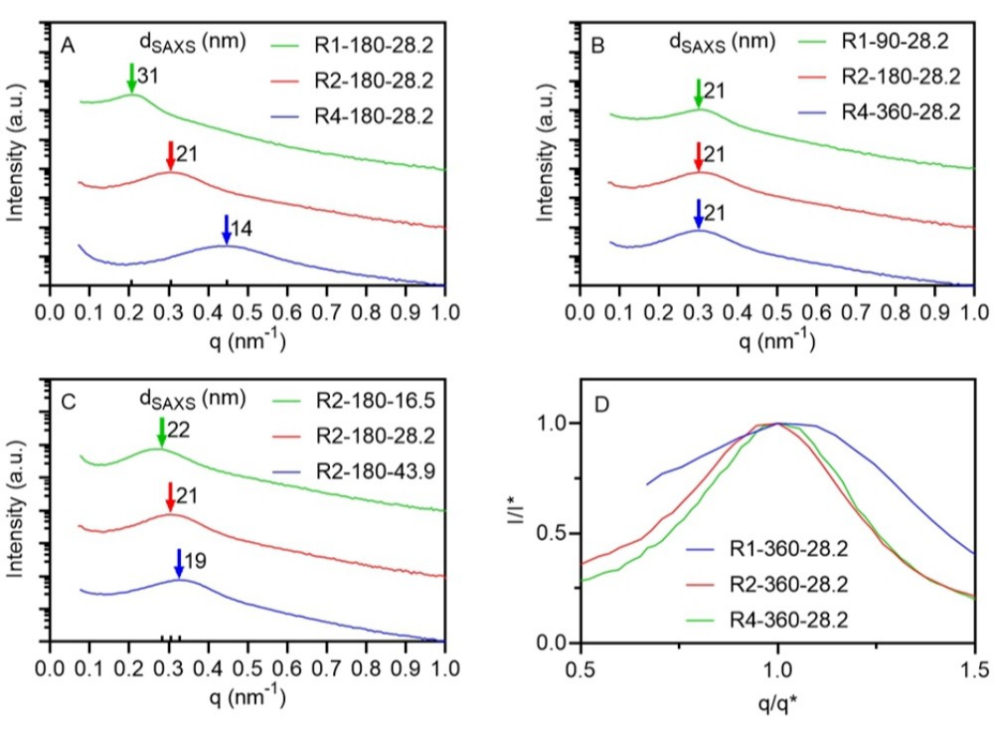
SAXS curves for materials 3D printed using: A) resins with 28.2 wt % of R1‐180 (green), R2‐180 (red), and R4‐180 (blue), B) resins with the *L*_ar*m*_=90 at the same macroCTA wt %, R1‐90‐28.2 (green), R2‐180‐28.2 (red), and R4‐360‐28.2 (blue), and C) resins with R2‐180 at 16.5 wt % (green), 28.2 wt % (red), and 43.9 wt % (green). D) Normalized SAXS profiles of materials 3D printed using resins with 28.2 wt % macroCTA, R1‐360‐28.2 (blue), R2‐360‐28.2 (red), and R4‐360‐28.2 (green).

Following the AFM study which revealed distinctive morphologies across 3D printed materials prepared with different resins, we conducted SAXS experiments to further explore the internal structure of the 3D printed materials. For the SAXS analysis, thin rectangular prisms (L×W×T=8×8×0.2 mm) were 3D printed using a layer cure time of 180 s and a layer thickness of 100 μm. As previously reported, the presence of a broad single maximum scattering intensity without higher‐order peaks in SAXS profiles reflects disordered microphase‐separated materials formed through the PIMS process.^[9a]^ Figure 6A compares the SAXS profiles of materials 3D printed using 28.2 wt % R1‐180, R2‐180, and R4‐180. The peak position q* (scattering vector *q* at maximum intensity) of R1, R2, and R4 were found at 0.21, 0.30, 0.45 nm^−1^, corresponding to *d*_SAXS_ of 31, 21, and 14 nm (Table 2). SAXS profiles of materials 3D printed using 28.2 wt % R1‐360, R2‐360, and R4‐360 also showed a similar trend of decreasing *d*_SAXS_ with decreasing *L*_ar*m*_ (SI, Figure S10). On the other hand, SAXS profiles of materials 3D printed using the resins R1‐90‐28.2, R2‐180‐28.2, and R4‐360‐28.2, which all have the same arm‐length (*L*_ar*m*_=90) and trithiocarbonate concentration, displayed the same q* at ∼0.3 nm^−1^, corresponding to a *d*_SAXS_ of 21 nm (Figure 6B). Additionally, materials 3D printed using the resins R1‐180‐28.2 and R2‐360‐28.2 with equal *L*_ar*m*_ of 180 also featured equal *d*_SAXS_ of 31 nm (SI, Figure S11). These results suggest that the domain spacing of 3D printed PIMS materials is mainly defined by the *L*_ar*m*_ of the macroCTA regardless of their architectures This finding is consistent with some previous reports of multi‐arm block copolymer self‐assembly, albeit under different processing conditions.^[28]^ Notably, however, other works have also observed smaller domain spacing for self‐assembled star block copolymers compared to linear block copolymer analogues.^[29]^ The comparison to the current work is difficult due to the different processing methods and chemical compositions, but regardless, the trends in the current work are clear, with the macroCTA *L*_ar*m*_ playing a more significant role in determining the domain spacing of 3D printed PIMS materials.

Upon increasing the macroCTA wt % from 16.5 to 43.9 wt % for the R2‐180 resins, the q* value of the SAXS profiles increased from 0.28 nm^−1^ to 0.33 nm^−1^ (Figure 6C), revealing a slight decrease in the domain spacing (*d*_SAXS_) from 22 nm to 19 nm. The decrease in *d*_SAXS_ is consistent with our previous study using linear (1‐arm) macroCTA, which was ascribed to reduction in average block copolymer size upon higher loading of macroCTA in a resin.^[24]^ Similarly, SAXS profiles of materials 3D printed using R4‐180 resins also displayed the same trend, with *d*_SAXS_ decreasing from 16 nm to 13 nm upon increasing macroCTA wt % from 16.5 to 43.9 wt % (SI, Figure S12). Importantly, the domain spacing values obtained by SAXS were in good agreement with the values determined by AFM (Table 2), which confirms that the AFM measurement provides a good representation of the internal structure. Overall, by defining the *L*_ar*m*_ of different macroCTAs, precise control over the nanostructure domain spacing was demonstrated.

Besides the domain spacing analysis, the sharpness of the compositional interfaces was also evaluated by comparing the breadth of the normalized SAXS profiles against the intensity (I*) and the position (q*) of the principal peaks. The majority of resins containing 28.2 wt % macroCTA resulted in 3D printed materials that exhibited similar normalized SAXS profiles, with the exception of the material prepared using R1‐360‐28.2 which gave an obviously broader peak. The broader peak indicates less sharp compositional interfaces for the R1‐360‐28.2 material compared to others (Figure 6D and SI, Figure S13).

For further examination, the SAXS results were fitted with the Teubner‐Strey (T−S) model.[^11a^, ^30^] The extracted T−S model parameters presented in SI, Table S2 shows that the domain spacing from SAXS and the T−S model (*d*_TS_) were in close agreement, thus confirming the suitability of T−S model for our system. In addition to the domain spacing, the T−S model provides structural information including the correlation length (*ξ*), the amphiphilicity factor (*f*_a_) and ratio of ξ/*d*_TS_, which is an indicator of domain size polydispersity.^[31]^ The *f*_a_ of the studied materials were typically between −0.88 and −0.90, suggesting well‐structured materials resulting from the PIMS process.^[11a]^ Interestingly, materials 3D printed using resins with macroCTA of similar *L*_ar*m*_ and macroCTA wt % exhibited extremely similar *f*_a_ and *ξ*/*d*_TS_ , indicating their similarity in terms of interfacial sharpness and domains size polydispersity (Table S2).^[31]^ As an exception, the material from R1‐360‐28.2 featured a relatively higher *f*_a_ of −0.77 and low *ξ*/*d*_TS_ of 0.44, indicating more diffuse domain interfaces and higher domain size polydispersity. The reduced domain definition in the R1‐360‐28.2 is likely related to the entanglements of the long arm of linear PBA‐macroCTA (R1) with *X_n_* of 360, which exceeds the critical entanglement length of PBA (approximately 25 kg mol^−1^).^[32]^

**Figure 7:**
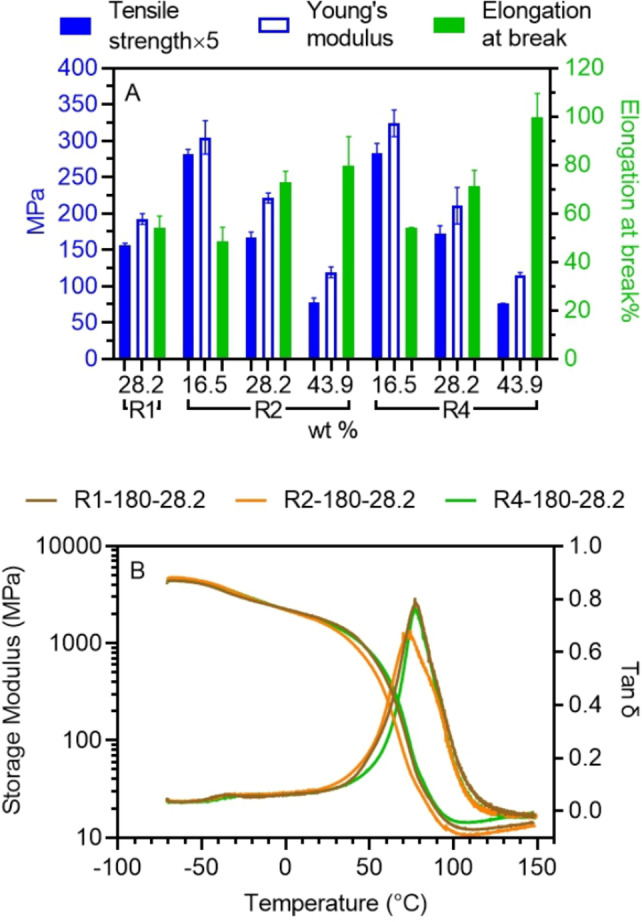
Tensile and thermomechanical properties of 3D printed materials. A) Tensile properties of materials 3D printed using different resins at fixed *X_n_* of 180. B) Storage modulus and tan δ profiles of materials 3D printed using resins containing macroCTAs at fixed *X_n_* of 180 with different architecture.

To investigate the influence of nanostructured morphology on bulk material mechanical properties, the tensile properties of 3D printed dumbbell‐shaped samples were evaluated at room temperature (∼23 °C). The obtained stress‐strain curves are presented in SI, Figures S14–S17. Interestingly, all resins produced materials with similar yield points at around 20 % strain but presented different elongations at break. Figure 7A summarizes the tensile properties of 3D printed PIMS materials prepared using resins containing varying amounts of different multi‐arm PBA‐macroCTAs at *X_n_* =180. In general, materials printed using resins with the same macroCTAs wt % displayed similar tensile strength and elongation at break values, even though their nanostructures, domain sizes and domain spacing were different. For instance, the tensile strength and elongation at break of 3D printed PIMS materials were in the range of 31–34 MPa and 54–73 % respectively, when using 28.2 wt % macroCTAs loading.

On the other hand, tunable tensile properties can be achieved by varying the macroCTA wt %. Specifically, the Young's modulus and tensile strength of 3D printed materials decreased, while elongation at break increased with increasing macroCTA wt %. For instance, increasing the loading of R2‐180 from 16.5 to 43.9 wt % in resins led to a reduction of tensile strength from 57 to 16 MPa and a reduced Young's modulus from 305 to 119 MPa for 3D printed PIMS materials, whereas, an increase of elongation at break from 48 to 80 % was observed (SI, Table S3). Despite significant changes in these mechanical properties, these materials displayed similar PBA domain width and domain spacing as evidenced by AFM and SAXS studies above.

The similarities in the mechanical properties for materials made with the same macroCTA loading was ascribed to the large contribution of the network phase to the overall material mechanical properties. Indeed, at a fixed loading of macroCTA, there is a fixed loading of AA and PEGDA, and thus a constant fraction of the network phase in the final materials. This behavior is consistent with previous reports of crosslinked materials,^[33]^ and differs from the mechanical property trends observed for thermoplastic elastomer systems, where the tensile properties are dependent on the material morphology and block copolymer architecture.^[34]^ In the latter case, ABA type star block copolymers present increased tensile strength than analogous AB type block copolymers due to the covalent linkage in the core the star.[^28b^, ^29^] In our case, the highly crosslinked network provides the majority of material strength.

To further examine the influence of nanostructure on the viscoelastic behavior of 3D printed PIMS materials, material thermomechanical properties were evaluated by dynamic mechanical analysis (DMA) in the range of −70 °C to 150 °C. Interestingly, despite their distinctive nanostructuration, using resins with macroCTAs of different architectures did not significantly affect the storage modulus (*G′*) and tan δ profiles of resulting 3D printed PIMS materials in cases when *X*_n_=180 (Figure 7B) or 360 (SI, Figure S18). Two distinct tan δ peaks at around −31 °C and 80 °C were observed in all cases, which were attributed to the glass transition of the PBA‐rich domain and the *net*‐P(AA‐*stat*‐PEGDA) domain, as previously reported.^[35]^ In contrast, the low temperature tan δ peaks were not found for materials prepared with analogous non‐PIMS resins containing BA monomer instead of PBA macroCTA (SI, Figure S19). Notably, the storage modulus of 3D printed PIMS materials underwent a great change when using resins with varying macroCTA wt % (SI, Figure S20A and C). For instance, the storage modulus at 25 °C decreased from 3 to 0.9 GPa when increasing the resin loading of R2‐180 from 16.5 to 43.9 wt %. The lower storage modulus at high macroCTA loading is due to the presence of more abundant soft PBA domains, contributing to increased network flexibility. As a result, the tan δ peaks, which is a ratio of the loss modulus to the storage modulus, at around −31 °C became more pronounced by increasing the macroCTA wt % (SI, Figure S20B and D). The thermomechanical properties observed by DMA are in accord with the tensile properties, which again verify the versatility of using different macroCTA architectures and wt % to independently control the nanostructure and mechanical properties of 3D printed materials.

**Figure 8:**
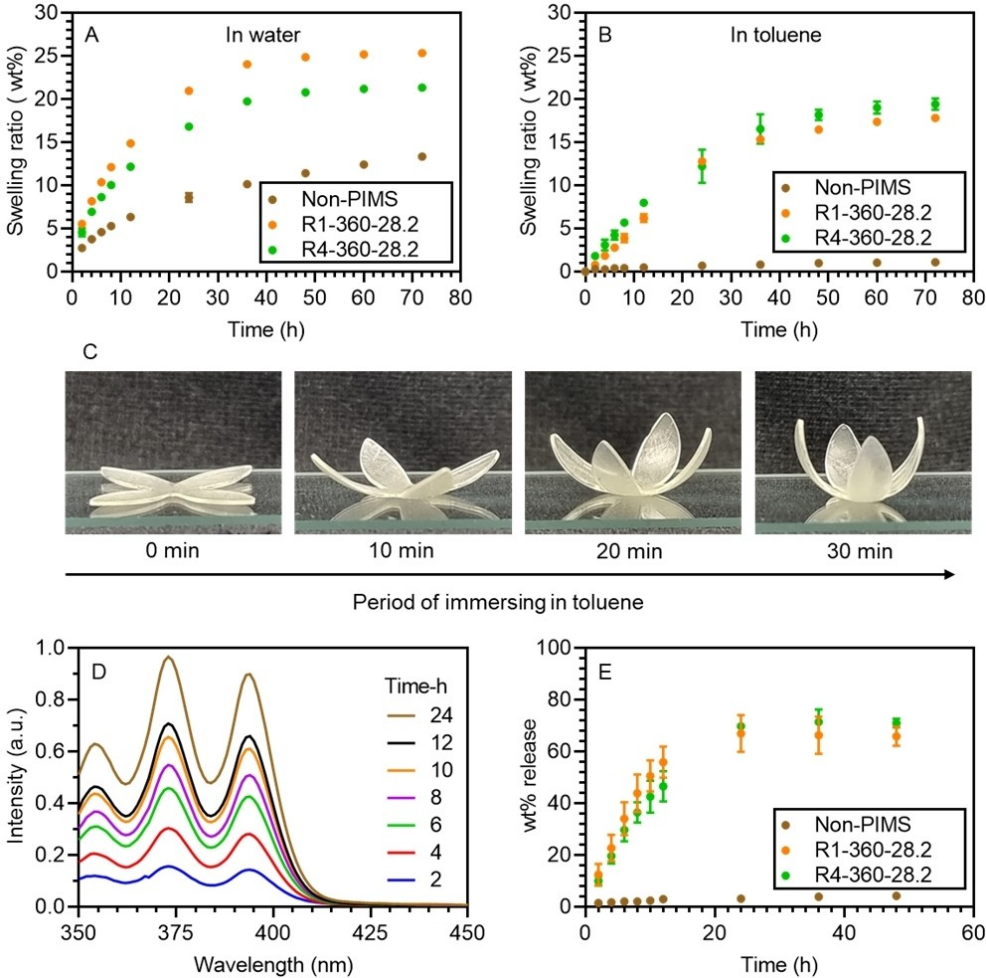
Swelling study of objects 3D printed using PIMS and non‐PIMS resins. Time dependent swelling in A) Water and B) Toluene. C) A flower‐shaped actuator 3D printed using PIMS resins (See Supporting Information for procedure). D) UV/Vis spectra of 9,10‐diphenylanthracene in toluene released by a square prism 3D printed using R1‐360‐28.2. E) wt % dye release in toluene over time for materials 3D printed using different resins. Note: A, B, and E: all experiments were performed in duplicate (for some values, the error bars were smaller than the data point).

The swelling behavior of polymer networks has drawn great attention in applications regarding solvent‐responsive actuation.^[36]^ However, the swelling behavior of PIMS materials in comparison with analogous non‐PIMS materials have not yet been investigated. To investigate the impact of material nanostructuration on the swelling behavior of 3D printed materials resins with and without macroCTA were selected to print samples with dimensions L×W×H=8×8×2 mm. Specifically, the resins selected for these swelling studies were R4‐360‐28.2, R1‐360‐28.2, which displayed interesting bicontinuous and phase‐inverted morphologies (Figure 5), as well as their non‐PIMS counterparts. Figure 8A shows the swelling behavior of 3D printed materials in water. Notably, the swelling rate and equilibrium swelling ratio of materials 3D printed using PIMS resins were significantly higher than non‐PIMS materials. For instance, 3D printing using R1‐360‐28.2 resulted in materials with a maximum swelling ratio in water of 25 wt % after 72 h, while the corresponding non‐PIMS material showed a swelling ratio of only 13 wt % after 72 h. Furthermore, non‐PIMS materials showed negligible swelling in toluene, while the swelling ratio of PIMS materials (R1‐360‐28.2) reached 18 wt % after 72 h (Figure 8B). The enhanced swelling behavior of the PIMS materials was attributed to the presence of well‐separated hydrophilic *net*‐P(AA‐*stat*‐PEGDA) and hydrophobic PBA domains, as evidenced by AFM (Figure 5C). Interestingly, materials prepared with R4‐360‐28.2 showed a slightly lower swelling ratio in water but higher swelling ratio in toluene compared with the material 3D printed using R1‐360‐28.2. This was scribed to the morphology of R4‐based materials which displayed more continuous hydrophobic PBA domains and less continuous hydrophilic P(AA‐*stat*‐PEGDA) domain (Figure 5I).

The observed dual‐affinity of the PIMS materials towards water and organic solvent could facilitate the design of complex swelling‐responsive functional parts via 3D printing. As a proof of concept, a flower‐shaped actuator was fabricated by successively printing with non‐PIMS and PIMS resins. Detailed fabrication procedures can be found in Supporting Information. Upon immersing the 3D printed multi‐material flower in toluene for 10 min, the flower underwent swelling induced actuation and bent to an observable degree due to the significant difference in the swelling rates of the non‐PIMS and PIMS portions of the material (Figure 8C). The degree of bending further increased with an extended period of immersion in toluene, reaching a highly curled structure after 30 min (Figure 8C). On the other hand, immersion in water for 30 min only resulted in a degree of actuation close to that observed in toluene for 10 min (SI, Figure S21). Extending the swelling time in water did not lead to further bending due to the intrinsically smaller difference in swelling rates and ratios between non‐PIMS and PIMS portions of the material.

Inspired by the high affinity of the PIMS materials for toluene, resins were loaded with 0.1 wt % of 9,10‐diphenylanthracene and used to 3D print square prisms (L×W×T=8×8×2 mm) for a model dye release study. The 3D printed prisms were immersed in 3 mL of toluene in a quartz cuvette with a stirrer bar to homogenize the solution. Figure 8D shows time‐resolved UV/Vis spectra of the dye release system for a prism prepared with the R1‐360‐28.2. The characteristic peaks for 9,10‐diphenylanthracene increased with time, indicating an efficient dye release from 3D printed PIMS samples. This efficient dye release was also observed for materials 3D printed using R4‐360‐28.2 (SI, Figure S22A). On the other hand, negligible dye release was observed for non‐PIMS materials over the same time scale (SI, Figure S22B), which agrees with the swelling results observed in toluene. Specifically, samples 3D printed using PIMS resins released more than 60 wt % of preloaded dyes and reached a plateau after 24 h, while the non‐PIMS counterparts showed only 4 wt % release (Figure 8E**)**.

## Conclusion

In conclusion, photocurable resins containing macroCTAs with different architectures were successfully applied, for the first time, for 3D printing of materials featuring ‘soft’ and ‘hard’ nanoscale domains. The size and morphology of the domains were readily controlled by tuning the structure and content of applied macroCTAs in the resin. In general, implementation of macroCTAs with longer arm length led to both increased PBA domain width and domain spacing as evidenced by AFM and SAXS analysis. In addition, Teubner‐Strey (T−S) modelling of SAXS data demonstrated similar interfacial sharpness and domains size polydispersity when macroCTAs with the same arm length were applied. Interestingly, implementation of resins containing multi‐arm macroCTAs enabled access to bicontinuous and phase‐inverted morphologies, while the corresponding monofunctional linear macroCTA‐based systems only resulted in bicontinuous morphologies. To our knowledge, this phase‐inverted morphologies morphology has not yet been observed in previous PIMS studies.

In terms of bulk material properties, the 3D printed PIMS materials displayed a wide range of tensile and thermomechanical properties by simply tuning the loading of different macroCTAs in the initial resins. No obvious dependence of these properties on macroCTA arm length and architecture was observed. As such, by tuning macroCTA arm length and architecture, different morphologies with varied domain spacing can be attained without significant influence on mechanical properties. Furthermore, 3D printed PIMS materials also featured a higher swelling ratio in both water and toluene than the non‐PIMS materials with analogous composition. The difference in swelling behavior was then leveraged to design a flower‐shaped material that showed different actuation in toluene and water. In addition, 3D printed PIMS materials were also shown to be effective for controlling dye release in toluene. These outstanding properties as well as access to different nanostructures is expected to open new opportunities in material design for 3D printing applications in various fields including soft actuators, biomedical devices, and advanced engineering structures.

## Conflict of interest

The authors declare no conflict of interest.

1

## Supporting information

As a service to our authors and readers, this journal provides supporting information supplied by the authors. Such materials are peer reviewed and may be re‐organized for online delivery, but are not copy‐edited or typeset. Technical support issues arising from supporting information (other than missing files) should be addressed to the authors.

Supporting Information

## Data Availability

The data that support the findings of this study are available in the supplementary material of this article.
