# Supplementary material for: Designing Nanostructured 3D Printed Materials by Controlling Macromolecular Architecture
Source: Angew Chem Int Ed Engl. 2022 Jul 18;61(35):e202206272. doi: 10.1002/anie.202206272 (PMC9544629; doi:10.1002/anie.202206272)
Supplement: Supplementary file 1 — Supporting Information [file ANIE-61-0-s001.pdf]

Angewandte Chemie
Eine Zeitschrift der Gesellschaft Deutscher Chemiker

Supporting Information

Designing Nanostructured 3D Printed Materials by Controlling Macromolecular Architecture

X. Shi, V. A. Bobrin, Y. Yao, J. Zhang, N. Corrigan*, C. Boyer**

Materials and Methods

Materials

The solvents were of either HPLC or AR grade; these included acetonitrile (RCI Labscan Limited, RCI Premium) and *N*,*N*-dimethylacetamide (DMAc, RCI Labscan Limited, HPLC). Aluminium oxide basic (Acros Organics, Brockmann I, 50–200 µm, 60A), 2-(*n*-butylthiocarbonothioylthio)propanoic acid (Boron Molecular, >95%), 3,5-bis(2-dodecylthiocarbonothioylthio-1-oxopropoxy)benzoic acid (Sigma-Aldrich, >98%), pentaerythritol tetrakis[2-(dodecylthiocarbonothioylthio)-2-methylpropionate] (Sigma-Aldrich, >97%), diphenyl (2,4,6-trimethylbenzoyl) phosphine oxide (TPO, Sigma-Aldrich, >97%), 2,2'-azobis(2-methylpropionitrile) solution (AIBN solution, Sigma-Aldrich, 0.2 M in toluene), acrylic acid (AA, anhydrous, Sigma-Aldrich, 99%) and poly(ethylene glycol) diacrylate (average M_n = 250, PEGDA, Sigma-Aldrich, >92%) were used as received. *n*-butyl acrylate (BA, Sigma-Aldrich, ≥99%) was passed through a basic aluminium oxide column to remove inhibitors before use. Unless otherwise stated, all chemicals were used as received.

Procedures for macroCTA synthesis

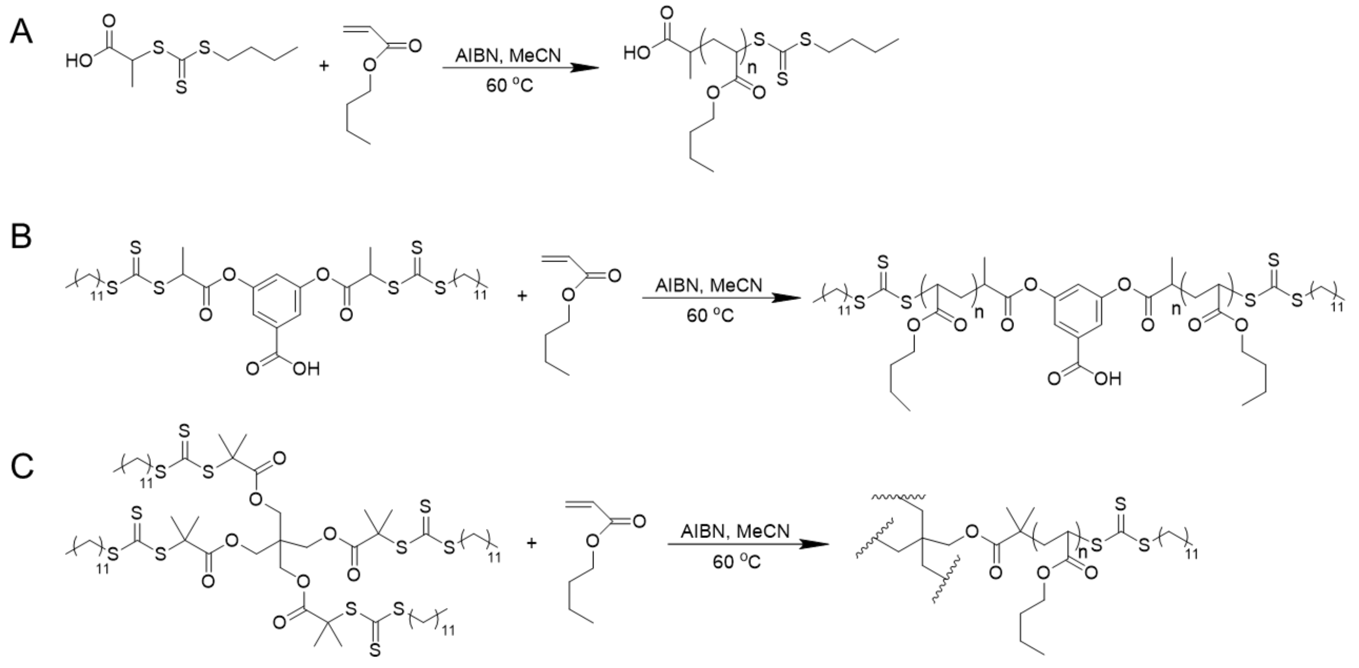

Scheme S1. Synthesis of PBA$_n$-macroCTA using RAFT polymerization of *n*-butyl acrylate. A) Synthesis of 1-arm PBA$_n$-macroCTA. B) Synthesis of 2-arm PBA$_n$-macroCTA. C) Synthesis of 4-arm PBA$_n$-macroCTA.

A typical procedure for the synthesis of the PBA$_n$-macroCTA is as follows: for the synthesis of R2-PBA$_{180}$-CTA, *n*-butyl acrylate (200 equiv., 50 g, 0.390 mol), 3,5-bis(2-dodecylthiocarbonothioylthio-1-oxopropoxy) benzoic acid (1 equiv., 1.60 g, 1.9×10^{-3} mol), and AIBN (0.1 equiv.,0.2 M solution in toluene, 0.97 mL, 1.9×10^{-4} mol) were dissolved in acetonitrile (99 mL). The mixture was deoxygenated by purging with nitrogen for 90 min and then polymerized for 15 h at 60°C. The reaction was stopped by cooling inside a freezer (-20°C) for 30 min and exposing to air. The polymer solution was concentrated by rotary evaporation and used without further purification. Using the same protocol, other PBA-CTAs were synthesized.

Resin preparation

Resins were prepared following the values reported in **Table S1**. The wt% of macroCTA were selected based on our previous study for potential comparison.[1] For instance, 10 g of resin R1-90-28.2 is consists of 2.82 g monofunctional linear macroCTA (X_n = 90), 3.82 g AA, 3.31 g PEGDA 250 and 0.05 g TPO. These components were vortex mixed for 30 s, followed by a 10 min bath sonication (Hwashin powersonic 410, 400 W). The obtained homogeneous mixture was then directly used for 3D printing. For non-PIMS resin counterparts, macroCTA was replaced by equivalent amount RAFT agent corresponding to 1-arm, 2-arm and 4 arm for R1, R2, R4 based resins respectively) and BA monomer.

Table S1: Summary of resin formulations in this study.

Resin formulations	PBA_n-macroCTA (wt%)	AA (wt%)	PEGDA250 (wt%)	TPO (wt%)
R1-90-28.2	28.2	38.2	33.1	0.5
R1-180-28.2	28.2	38.2	33.1	0.5
R1-360-28.2	28.2	38.2	33.1	0.5
R2-180-16.5	16.5	44.5	38.5	0.5
R2-180-28.2	28.2	38.2	33.1	0.5
R2-180-43.9	43.9	29.8	25.8	0.5
R2-360-28.2	28.2	38.2	33.1	0.5
R4-180-16.5	16.5	44.5	38.6	0.5
R4-180-28.2	28.2	38.2	33.1	0.5
R4-180-43.9	43.9	29.8	25.8	0.5
R4-360-28.2	28.2	38.2	33.1	0.5

3D printing procedures

Unless specifically mentioned, all samples for AFM, SAXS, DMA, tensile testing, and the swelling study were 3D printed using a DLP 3D printer (Anycubic Photon S) with a violet light LED array (λ_{max} = 405 nm, I_0 = 0.4 mWcm^{-2}). Samples were 3D printed using a layer thickness of 100 μm and curing time per layer of 180 s. The printed objects were separated from the build stage, washed with ethanol, air dried, and post-cured under violet light (λ_{max} = 405 nm, 10 mWcm^{-2}) for 15 min before any measurement.

The flower-shaped solvent responsive actuator (0.6 mm in thickness) was 3D printed using a layer thickness of 100 μm and curing time per layer of 180 s. The 3D printing program was paused after finishing printing of three layers using non-PIMS counterpart resin (10g, weight ratio of BA:AA:PEGDA:TPO:BTPA = 27.7 : 38.2 : 33.1 : 0.5 : 0.55) of R1-360-28.2. Afterward, a resin vat filled with R1-360-28.2 resin was replaced on the printer and the printing program was restarted to finish up the printing of the rest 3 layers. The printed flower was separated from the build stage, washed with ethanol, air dried, and post-cured under violet light (λ_{max} = 405 nm, 10 mWcm^{-2}) for 15 min before any measurement.

The cubic lattice structure was designed using Fusion 360 modelling software with dimension L × W × T = 30× 30 × 30 mm and rectangular strut of 0.9 mm in width. The designed model was then 3D printed with the cure time per layer of 25 s and layer thickness of 100 μm using Anycubic Photon Mono X 3D printer (λ_{max} = 405 nm, I_0 = 0.9 mWcm^{-2}).

Swelling study and swelling-induced actuation of 3D printed flower

3D printed rectangular prisms (L × W × T = 8 × 8 × 2 mm) were examined to determine the swelling properties in water and toluene of materials fabricated using various resins. The rectangular prisms were weighed before swelling to provide W_0. Then, they were immersed in 5 mL of water or toluene in 20 mL glass vials for swelling. At fixed time intervals, the samples were weighed after removing the excess solvent by wiping with paper towel to get W_t. The wt% swelling ratios were then calculated by the following **Equation 1**:

$$Swelling\ ratio(\%) = 100 \times \frac{W_t - W_0}{W_0} \tag{1}$$

The printed and post-cured multi-material flower was put into a beaker filled with toluene or water, with the PIMS-part of the material facing down. The flower was taken out of the beaker at 10 min increments, pictures were taken, and the flower was returned to the beaker.

Model dye release experiments with 3D printed materials

The studied resins were loaded with 0.1 wt% of 9,10-diphenylanthracene prior to 3D printing. Rectangular prisms (L × W × T = 8 × 8 × 2 mm) were 3D printed using a layer thickness of 100 μm and curing time per layer of 180s. The 3D printed prisms were washed with ethanol, air dried, and post-cured under violet light (λ_{max} = 405 nm, 10 mWcm^{-2}) for 15 min before any measurement. The post-cured rectangular prisms were weighed to calculate the weight of loaded dyes (0.1 × measured weight / 100). For UV-Vis. monitoring of dye release, the dye-loaded materials were placed in individual quartz cuvettes (1 × 1 cm) with 3 mL of toluene. Then, a stir bar was introduced to the cuvette to facilitate fluid movement and reduce concentration gradients in the cuvette. UV-Vis spectra of 9,10-diphenylanthracene in toluene released by 3D printed dye-loaded samples were obtained at several time points to allow calculation of the dye release profiles. The concentration of dye in toluene was calculated from a calibration curve of 9,10-diphenylanthracene based on its absorbance at 375 nm.

Characterizations

Nuclear magnetic resonance (NMR)

All NMR spectra were recorded on Bruker Avance III 400 MHz spectrometer using an external lock ($CDCl_3$). Monomer conversion was calculated by comparing integrals of residual monomers (at ~ 6 ppm) and polymers (at 4.05 ppm, a in Figure S2) in NMR spectra (**Figure S2**). $M_{n,theo.}$ was calculated based on monomer conversion determined by NMR as $M_{n,theo}$ = ([BA]$_0$/[RAFT agent]$_0$) × conv. (BA) × MW(BA) + MW(RAFT agent). The degree of polymerization (X_n) of PBA-CTAs was calculated based on the integral value at 4.05 ppm (the peak a in Figure S2) normalized against the peak for RAFT end group (at 4.8 ppm, c in Figure S2), $X_n = I_{4.05}/2$. $M_{n,NMR}$ was calculated as $M_{n,NMR} = X_n$(PBA-CTA) × MW(BA) + MW(RAFT agent). End group fidelity was calculated using the following equation: f (%) = $I_{4.8}/(I_{3.35/2})$ × 100%, where $I_{3.35}$ and $I_{4.8}$ are integral values at 3.35 and 4.8 ppm, respectively, which represent the peaks c and d in Figure S2.

Size exclusion chromatography (SEC)

Molecular weight distributions for all polymers were obtained via SEC using a Shimadzu modular system composed of an SIL-20A auto-injector, a Polymer Laboratories 5.0 μm bead-size guard column (50 × 7.5 mm^2) followed by three linear PL (Styragel) columns (10^5, 10^4 and 10^3), an RID-10A differential refractive-index (RI) detector. The eluent was DMAc (containing 0.03% w/v LiBr and 0.05% w/v 2,6-dibutyl-4-methylphenol (BHT)) at 50°C, run at a flow rate of 1.0 mL/min. The SEC system was calibrated using narrow polystyrene (PSTY) standards with molecular weights of 200 – 10^6 g/mol.

Attenuated total reflectance - Fourier transform infrared (ATR-FTIR) spectroscopy for monitoring polymerization kinetics

ATR-FTIR spectroscopy was performed to monitor photopolymerization kinetics using a Bruker Alpha FTIR spectrometer equipped with room temperature DTGS detectors. After taking a background reading of the empty plate, 20 μL of polymerization resin was pipetted onto the ATR crystal plate. An absorption spectrum was then obtained by scanning the droplet from 400-4000 cm^{-1}. After an initial reading, the IR absorption spectra were obtained at various times of irradiation with a Thorlabs mounted LED with a collimation adapter (λ_{max} = 405 nm, I_0 = 3.7 mW cm^{-2}). The vinyl bond conversion was deduced from the disappearance of the C=C bending peak at 1630 cm^{-1} normalized to the C=O stretching peak at 1760 cm^{-1} as an internal standard.

As such, the real-time conversion was calculated by **Equation 2**, where α_t is vinyl bond conversion after irradiation of t s, int_0 and std_0 refer to the integration of peak in the range of 1600-1650 cm^{-1} and 1670-1800 cm^{-1} for unreacted sample, int_t and std_t refer these two values after irradiation of t s

$$\alpha_t = 1 - (int_t/std_t)/(int_0/std_0) \quad (2)$$

UV-Vis spectroscopy

All UV-vis spectra were recorded using a Varian Cary 300 spectrophotometer. Scans were conducted in the range of 600-200 nm at 600 nm/min. Resin measurement and dye release experiments were conducted in a 1×1 cm quartz cuvette. A spectrum obtained with an empty cuvette was used for zero/baseline correction.

Atomic force microscopy (AFM)

All AFM measurements were performed on the Bruker Dimension ICON SPM, with a NanoScope V controller (software version 9.70). Mechanical property mapping was performed using peak force tapping mode on the top layer of printed objects using the SCANASYST probe (from Bruker AFM probes). The scan size was set to 1 µm. The scan rate was set at around 0.7 to 0.8 Hz with a peakforce of approximately 500 pN. The feedback gain was adjusted accordingly to optimize tracking of the specimen surface, without any significant feedback noise. The resolution of the image was set to 512 pixels per line for a 1 µm scan size. For peakforce QNM measurements, the tip was calibrated using the thermal tunning method. AFM images were analyzed using NanoScope Analysis software, version 1.7. For the statistical length analysis, at least 50 areas were carefully traced by hand to determine average domain size and domain spacing using ImageJ software. Histograms of the size distribution were constructed. Average PBA domain width (*D*), domain spacing (*d*) were calculated using **Equation 3**:

$$d_n = \frac{\sum_{i=1}^{n} N_i d_i}{\sum_{i=1}^{n} N_i} \quad (3)$$

Where *N* is the number of observations and *d* is the determined size for each measurement. *d* was defined as the center-to-center distance of the two PBA domains/phases. *D* and *d* were defined according to the schematic picture below.

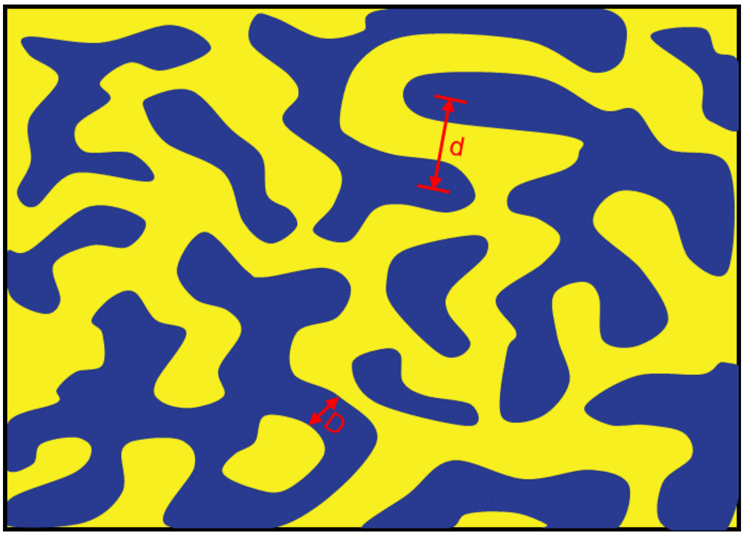

Scheme S2. Schematic illustration of microphase-separated morphology. PBA domains are shown in blue; *net*-P(AA-*stat*-PEGDA) domains are shown in yellow. D – PBA domain width (D_{PBA}); d – domain spacing (d_{AFM}).

Small-angle X-ray scattering (SAXS)

SAXS experiments were performed on an Anton Paar SAXSPoint 2.0 system with a Cu K_{α} (λ = 0.154 nm) microfocus X-ray source (50 kV/1 mA) and Dectris Eiger 1M detector. Data were collected at room temperature, under vacuum for 5 min from a sample at a sample-to-detector distance of 0.575 m. Samples were 3D printed using a thickness of 100 μm and 2 layers. Data was reduced to 1D by radial averaging the 2D detector after converting pixel positions to $q = (4\pi/\lambda)\sin\theta$, where 2θ is the scattering angle. The domain spacing was calculated using **Equation 4**, where q is the scattering vector:

$$d_{\text{SAXS}} = \frac{2\pi}{q} \qquad (4)$$

SAXS fitting using Teubner-Strey (T-S) model

The SAXS data for microphase-separated 3D printed materials were fitted using T-S model[2] in SasView software. According to T-S model

$$I(q) = \frac{1}{a_2 + c_1 q^2 + c_2 q^4} + b \qquad (5)$$

Where $q = (4\pi/\lambda)\sin\theta$, λ is the wavelength, 2θ is the scattering angle; b is background scattering; a_2, c_1, c_2 are fitting parameters used to calculate domain spacing (d_{TS}), correlation length (ξ) and the amphiphilicity factor (f_a) using **Equations 6-8** below:

$$d_{TS} = 2\pi\left[\frac{1}{2}\left(\frac{a_2}{c_2}\right)^{1/2} - \frac{1}{4}\frac{c_1}{c_2}\right]^{-1/2} \quad (6)$$

$$\xi = \left[\frac{1}{2}\left(\frac{a_2}{c_2}\right)^{1/2} + \frac{1}{4}\frac{c_1}{c_2}\right]^{-1/2} \quad (7)$$

$$f_a = \frac{c_1}{\sqrt{4a_2c_2}} \quad (8)$$

Dynamic mechanical analysis (DMA)

Dynamic mechanical analysis (DMA) of 3D printed specimens was conducted using single cantilever mode on a TA Q800 dynamic mechanical analyzer. Specimens were 3D printed with the designed dimension of l × w × t = 40 × 8 × 2 mm. The actual dimensions were measured with a digital calliper. The experimental method was applied for all specimens as follows: equilibrate at -70°C, isothermal for 3 min, temperature ramp to 150 °C at 2 °C/min, constant frequency of 1 Hz and displacement of 15 µm. The glass transition temperature (T_g) was determined using the temperature at the peak of the tan δ curve.

Tensile testing

Dog-bone specimens were designed using Fusion 360 3D modelling software by modifying the ASTM D638 Type I specimen[3] and the object was exported as an .stl file. Specimen dimensions were: thickness (T) = 2.04 mm, width overall (W_O) = 8.38 mm, length overall (L_O) = 50.3 mm, distance between grips (D) = 36 mm, gauge length (G) = 15.79 mm, width at the center (W_c) = 6 mm.

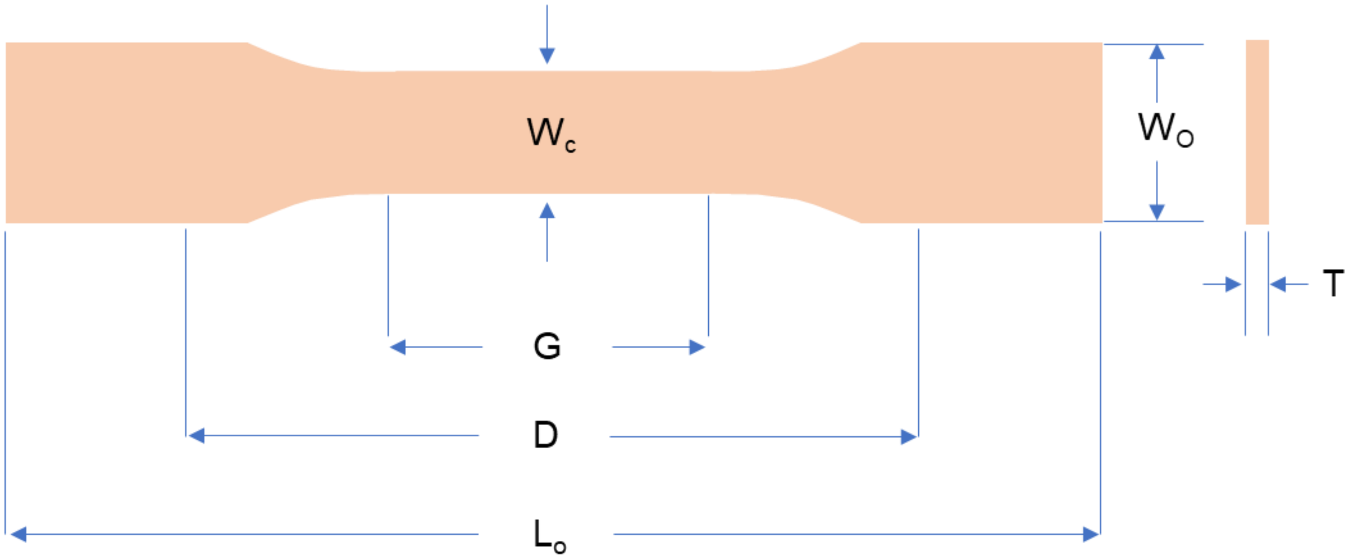

The tensile tests were performed using a Mark–10 ESM303 with a 1 kN force gauge. The travel speed for all tests was 1.1 mm/min. All tensile results were performed in triplicate. The tensile stress (MPa) was calculated

as the maximum force (N) divided by the initial cross-sectional area (mm^2) of the gauge section. The strain was determined as the measured travel distance relative to the original specimen gage length, expressed as a percentage.

Additional Data

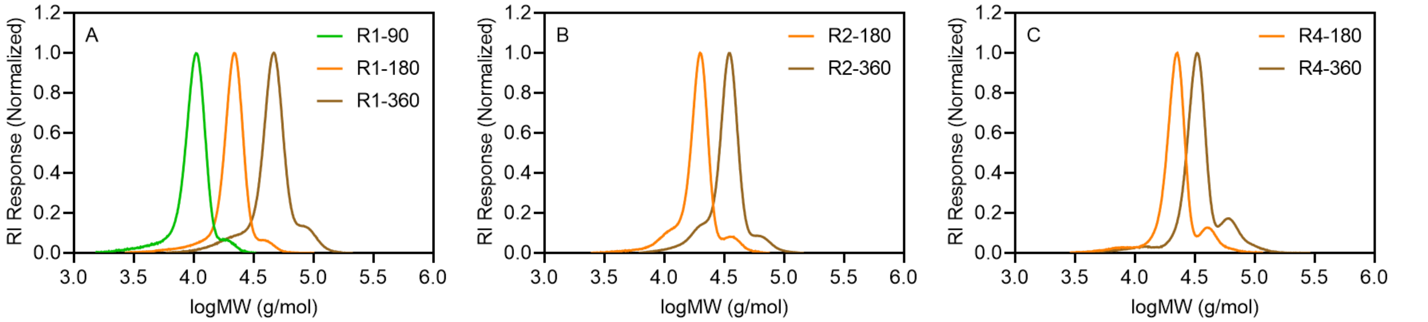

Figure S1: Molecular weight distributions for PBA$_n$-macroCTAs obtained by SEC. A) 1-arm PBA$_n$-macroCTAs with targeted overall degree of polymerization (X_n) of 90, 180, and 360. B) 2-arm PBA$_n$-macroCTAs with targeted X_n of 180, and 360. C) 4-arm PBA$_n$-macroCTAs with targeted X_n of 180, and 360.

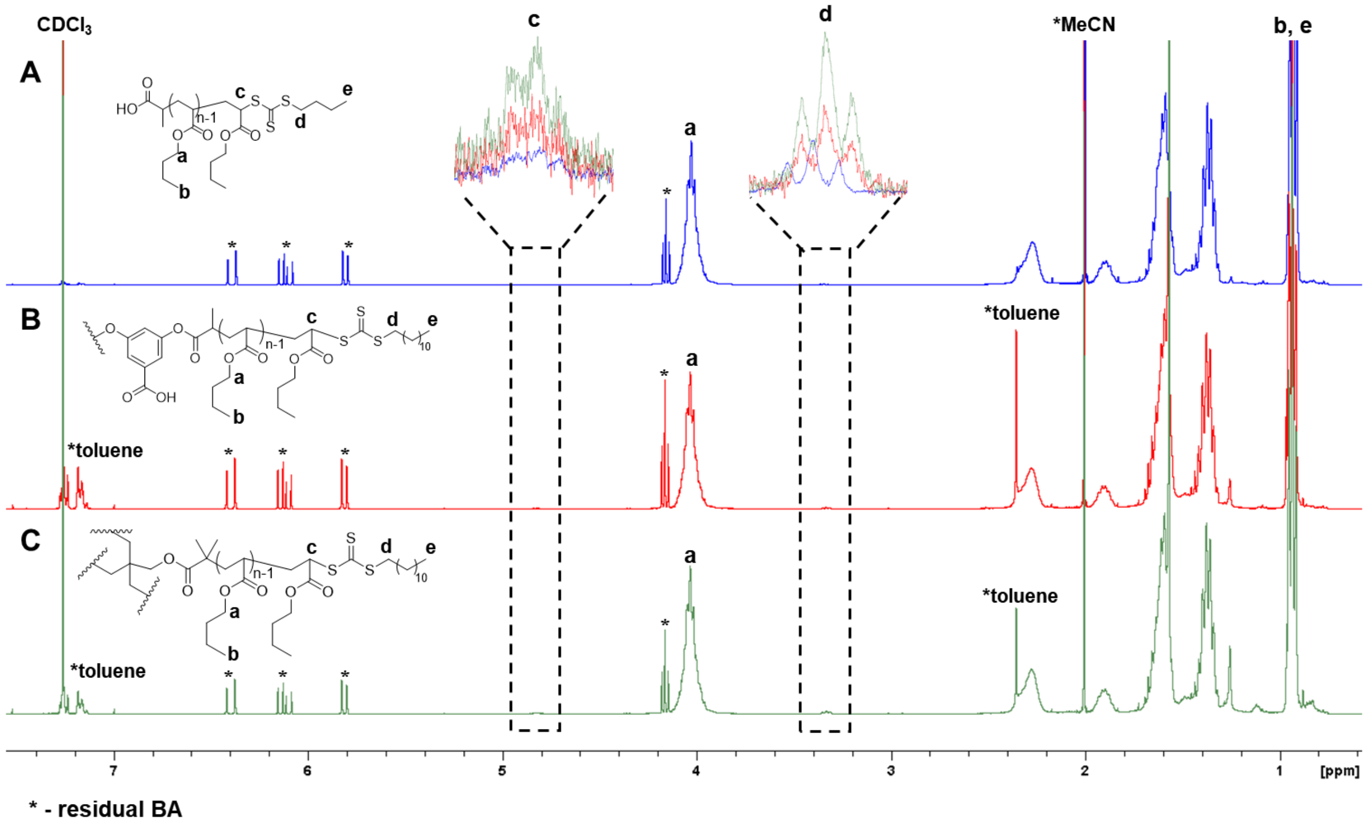

Figure S2: ^{1}H NMR spectra of synthesized PBA-macro-CTAs with different architectures. A) 1-arm PBA-macro-CTA with overall degree of polymerization (X_n) of 360. B) 2-arm PBA-macro-CTA with overall degree of polymerization (X_n) of 360. C) 4-arm PBA-macro-CTA with overall degree of polymerization (X_n) of 360. Recorded using ^{1}H NMR spectra (400 MHz, $CDCl_3$, 298 K). Note: * - residual BA monomer and residual acetonitrile (MeCN). The integrals were normalized by using signal at 4.03 ppm. See SI, Characterization, NMR section for detailed procedure for calculation of X_n and $M_{n,\,NMR}$.

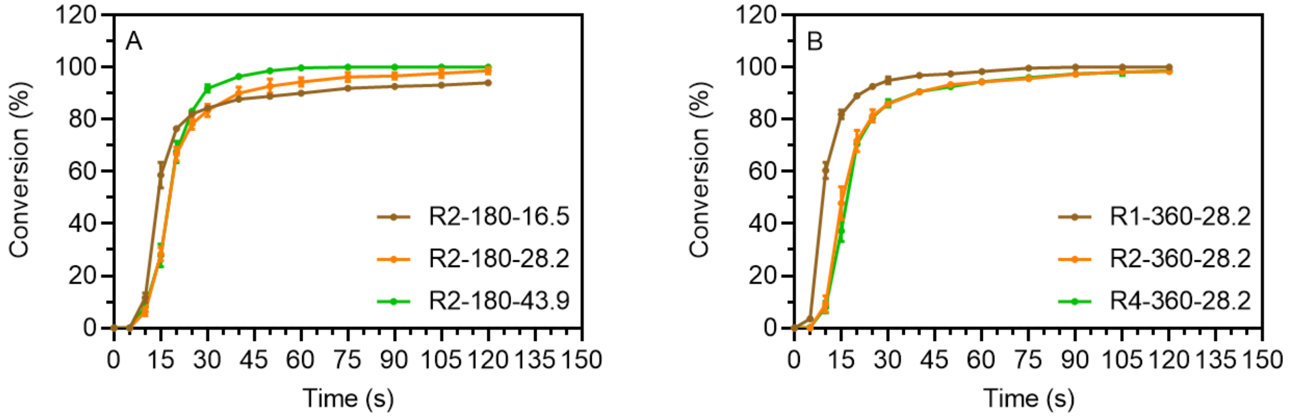

Figure S3: Polymerization kinetics for photocurable resins under violet light irradiation (405 nm, 3.7 mW cm^{-2}). A) Resins containing different weight ratios (16.5, 28.2, and 43.9 wt%) of 2-arm macroCTA (X_n = 180), B) Resins containing 28.2 wt% of 1-arm, 2-arm and 4-arm macroCTAs (X_n = 360).

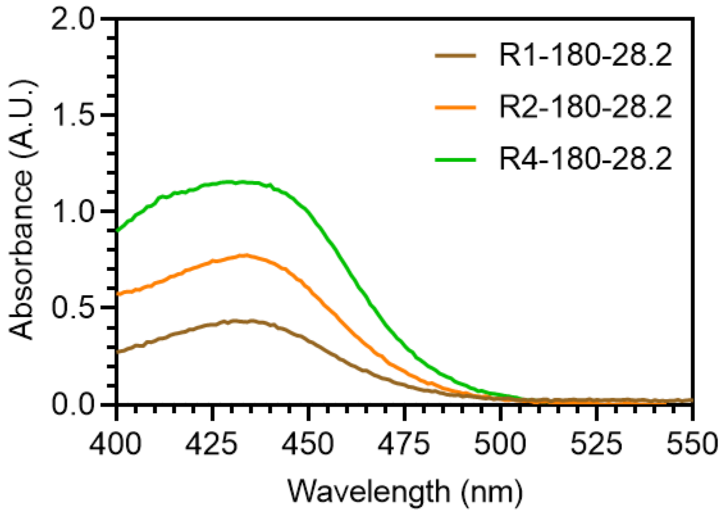

Figure S4: UV-Vis. spectra of resins with 28.2 wt% of R1 (brown line), R2 (orange line), and R4 (green line), respectively.

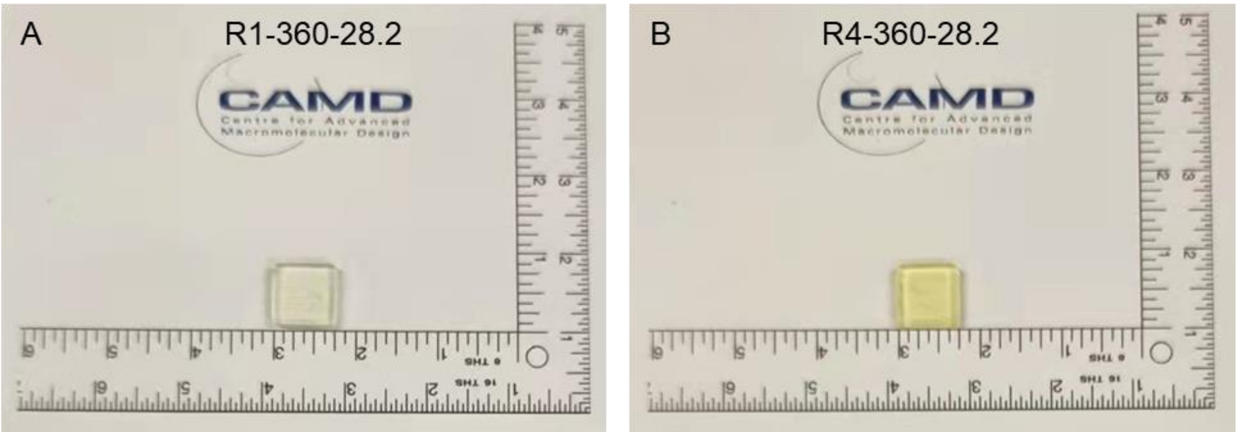

Figure S5: 3D printed rectangular prism (L × W × T = 8 × 8 × 2 mm) for printing condition test prepared using resin A) R1-360-28.2 and B) R4-360-28.2. A commercially available 3D printer (Anycubic PhotonS, λ_{max} = 405 nm (violet light), I_0 = 0.4 mWcm-2) was used with a layer thickness of 100 µm and curing time per layer of 180 s.

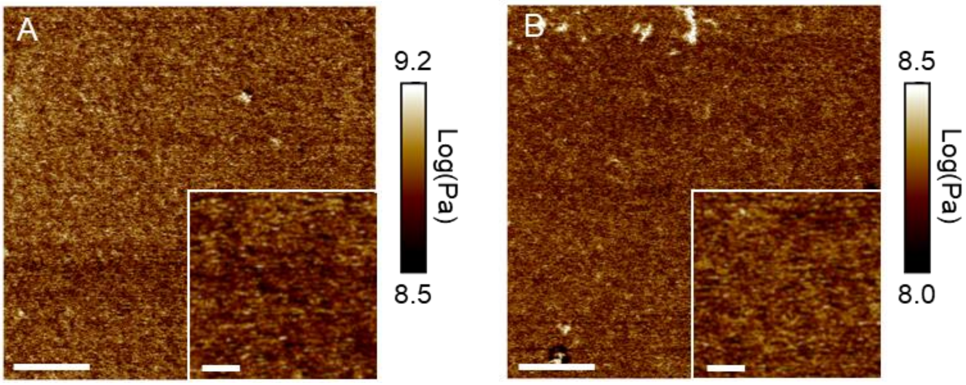

Figure S6: Surface morphologies of materials 3D printed using non-PIMS resins with BA used in place of PBA: counterpart of A) R2-180-28.2 and B) R4-180-28.2. Inset: Magnified views of AFM images, Scale bar is 200 nm for main images and 40 nm for insets.

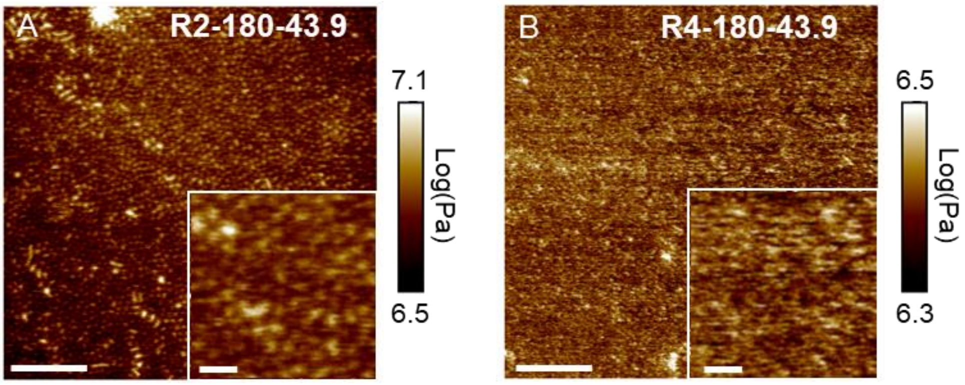

Figure S7: Surface morphologies of materials 3D printed using PIMS resins: A) R2-180-43.9 and B) R4-180-43.9. Inset: Magnified views of AFM images, Scale bar is 200 nm for main images and 40 nm for insets. D_{PBA} and d_{AFM} were not calculated for these two samples due to the poor image quality.

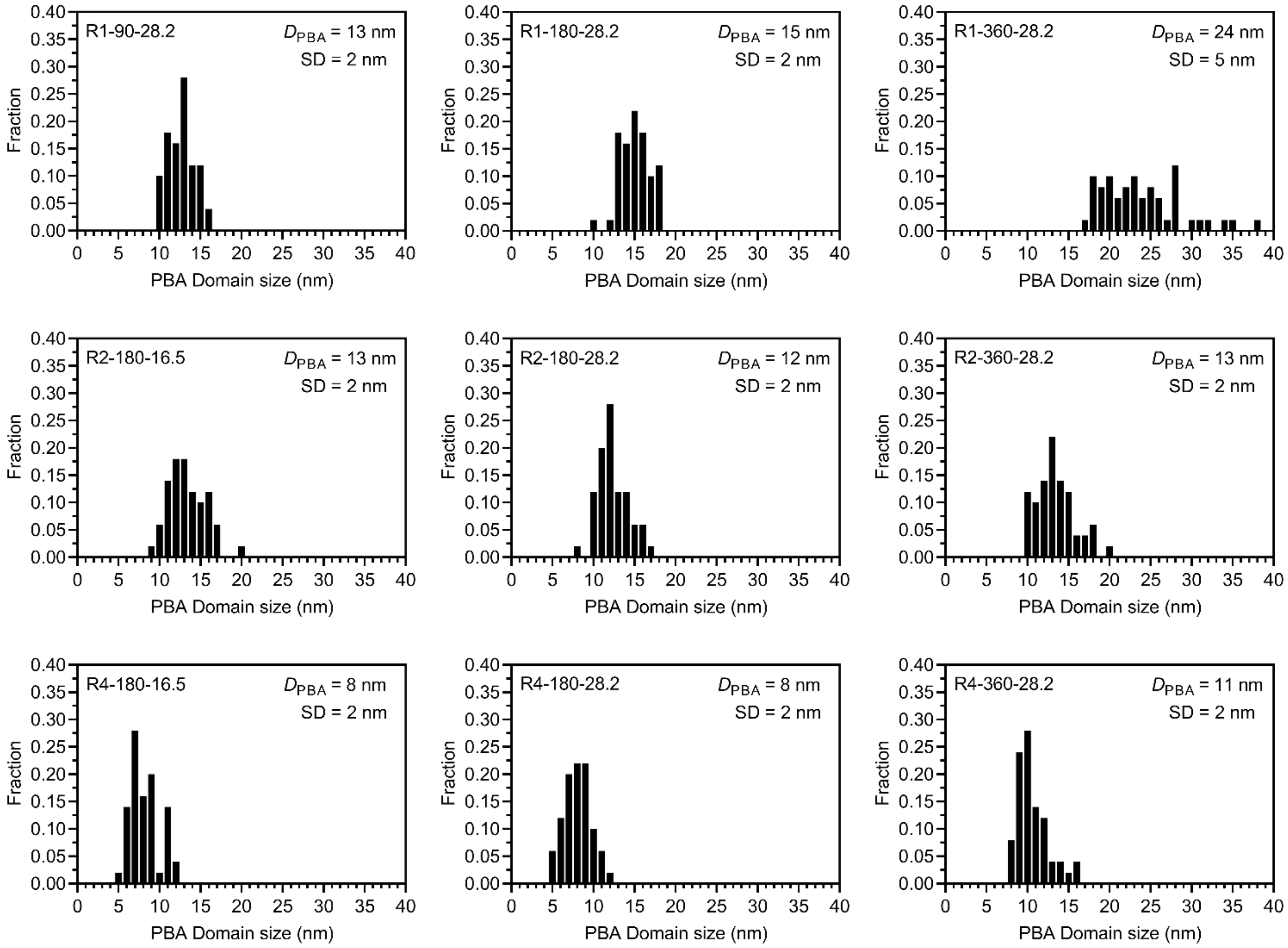

Figure S8: Histograms of PBA domain sizes of 3D printed materials. See Atomic force microscopy (AFM) and scheme S2 in characterization section for calculation procedure.

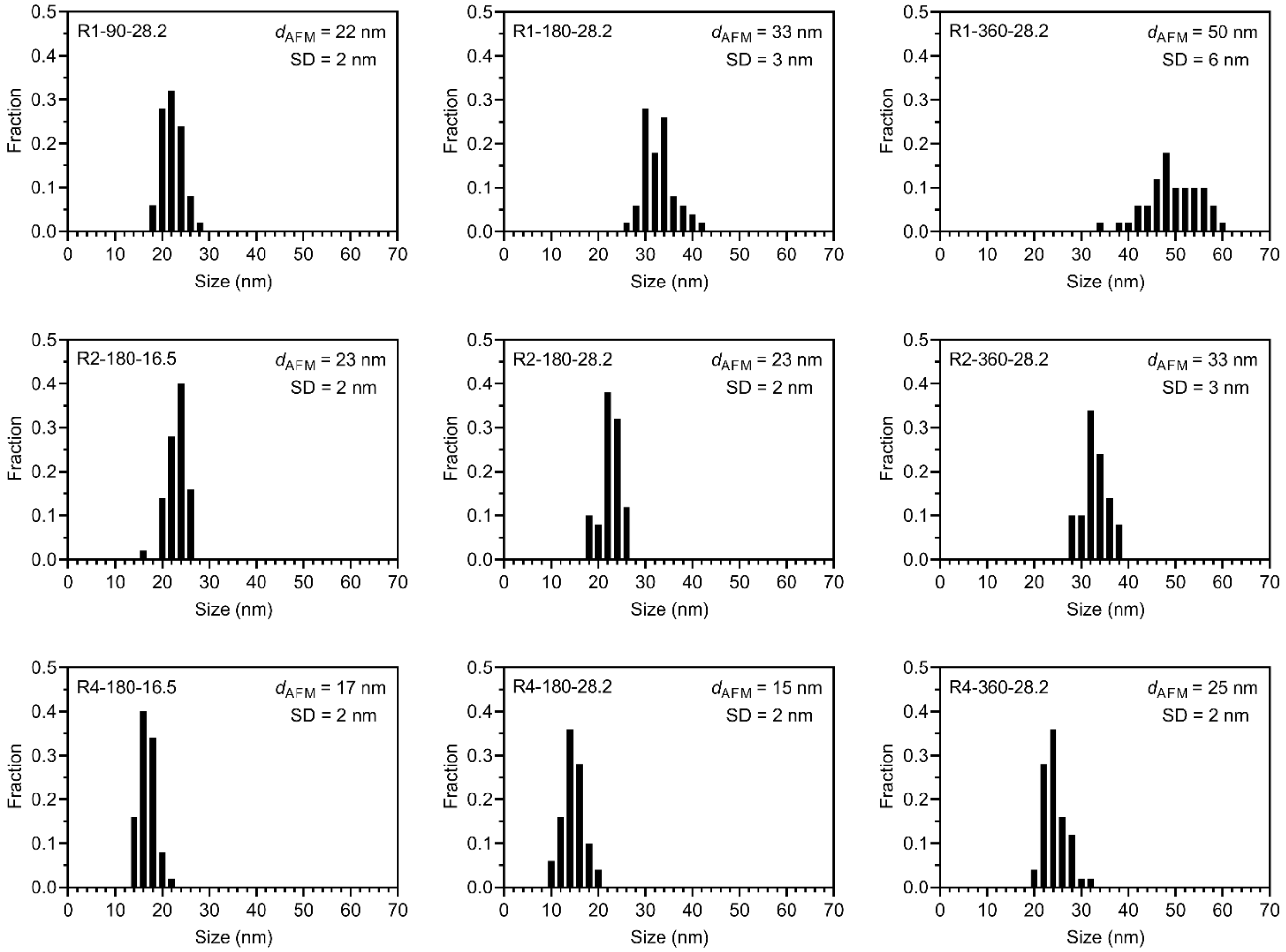

Figure S9: Histograms of domain spacings of 3D printed materials. See atomic force microscopy (AFM) and scheme S2 in characterization section for calculation procedure.

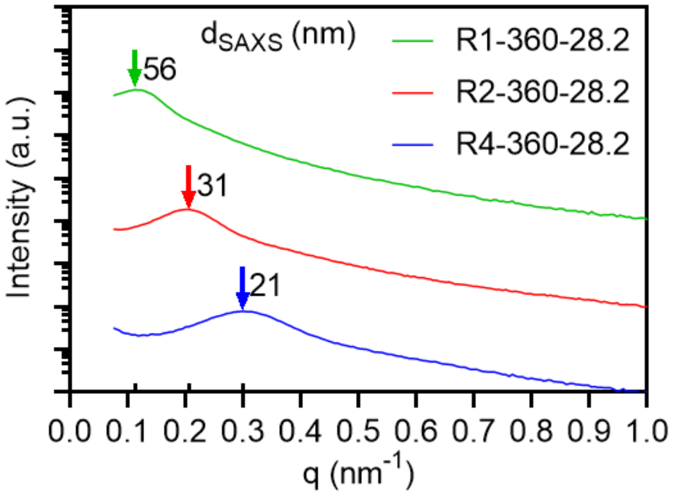

Figure S10: SAXS profiles of materials 3D printed using resins containing 28.2wt% of 1-arm, 2-arm, and 4-arm macroCTAs (X_n = 360) respectively.

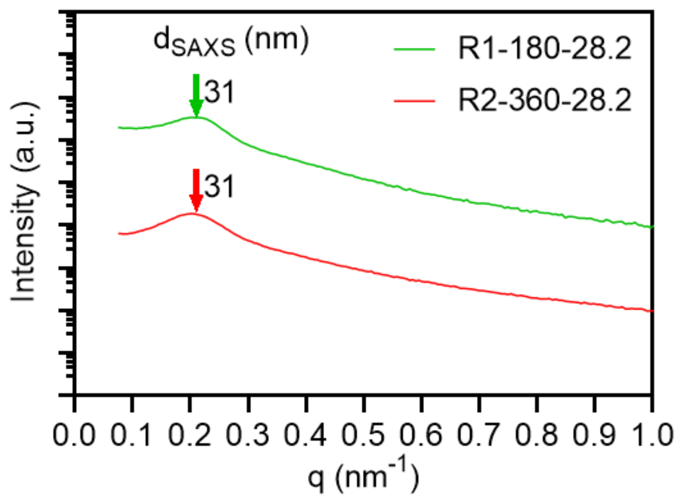

Figure S11: SAXS profiles of materials 3D printed using resins containing 28.2wt% of 1-arm and 2-arm macroCTAs respectively with the same arm length (L_{arm} = 180).

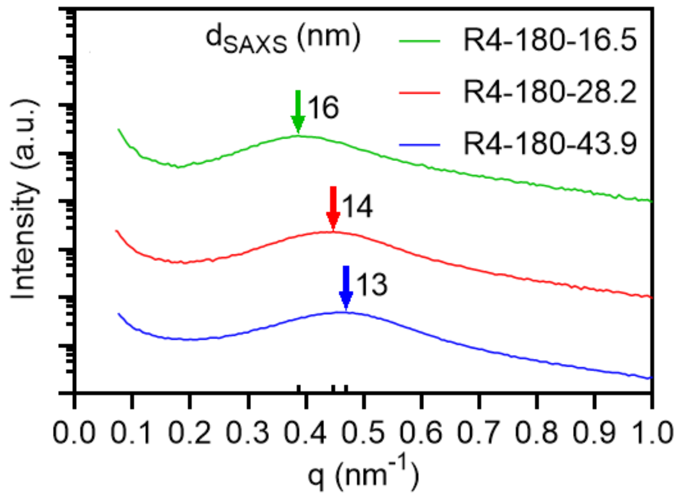

Figure S12: SAXS profiles of materials 3D printed using resins containing different weight ratios of 4-arm macroCTAs (X_n = 180).

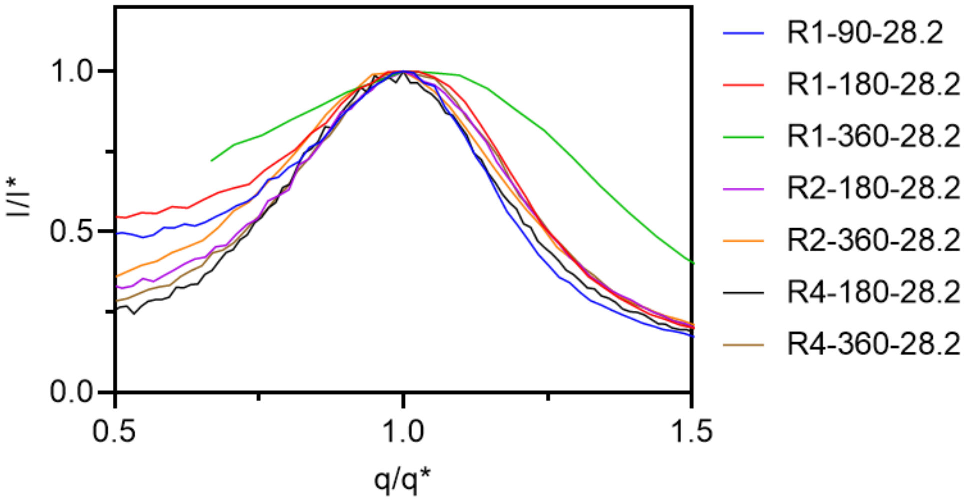

Figure S13: Normalized SAXS profiles for peak breadth comparison for thin rectangular prisms 3D printed using resins with 28.2 wt% of 1-arm, 2-arm and 4-arm macroCTA at different X_n.

Table S2. Parameter values obtained from fitting of SAXS peaks using Teubner-Strey (T-S) model.

MacroCTA architecture	Formulas	X_n	a_2[b]	c_1[b]	c_2[b]	d_{SAXS} (nm)[c]	d_{TS} (nm)[d]	ξ (nm)[e]	ξ/d_{TS}[f]	f_a[g]
1 arm	R1-90-28.2	94	439.8	-7980.3	44241.7	21.0	20.4	14.5	0.71	-0.90
	R1-180-28.2	180	321.9	-11934.6	140134.3	31.0	29.5	19.3	0.65	-0.89
	R1-360-28.2	360	76.9	-6743.6	248141.2	56.0	50.3	22.3	0.44	-0.77
2 arm	R2-180-16.5	180	282.1	-5812.0	38580.9	22.0	22.1	14.0	0.63	-0.88
	R2-180-28.2	180	421.9	-7207.7	37783.3	21	19.8	13.9	0.70	-0.90
	R2-180-43.9	180	322.4	-4739.9	22059.1	19.0	18.6	12.2	0.66	-0.89
	R2-360-28.2	340	296.6	-11171.0	134625.9	31.0	29.9	19.2	0.64	-0.88
4 arm	R4-180-16.5	186	308.0	-3051.6	9626.2	16.0	15.3	9.9	0.65	-0.89
	R4-180-28.2	186	391.6	-3184.3	8010.1	14.0	13.7	9.5	0.69	-0.90
	R4-180-43.9	186	354.6	-2649.6	6195.1	14.0	13.2	8.9	0.67	-0.89
	R4-360-28.2	360	377.7	-6609.6	35932.2	21.0	20.1	13.8	0.69	-0.90

[a] – the degree of polymerization of PBA-CTA; [b] – parameters calculated from SAXS fitting using T-S model; [c] – domain spacing determined from SAXS; [d] – domain spacing determined from T-S fitting using equation S5; [e] – correlation length determined from T-S fitting using equation S6; [f] – The ratio of ξ/d_{TS} is a measure of the domain size polydispersity, the smaller the ratio, the larger the polydispersity[4]; [g] – amphiphilicity factor determined using equation 8.

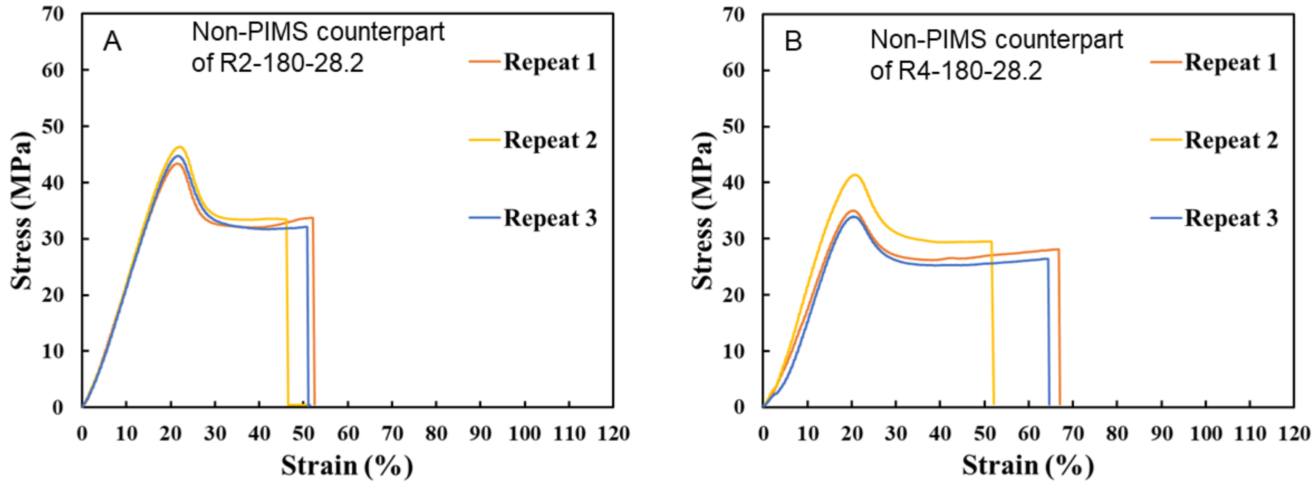

Figure S14: Stress-strain curves of objects 3D printed using resin: A) Non-PIMS counterpart of R2-180-28.2, B) Non-PIMS counterpart of R4-180-28.2.

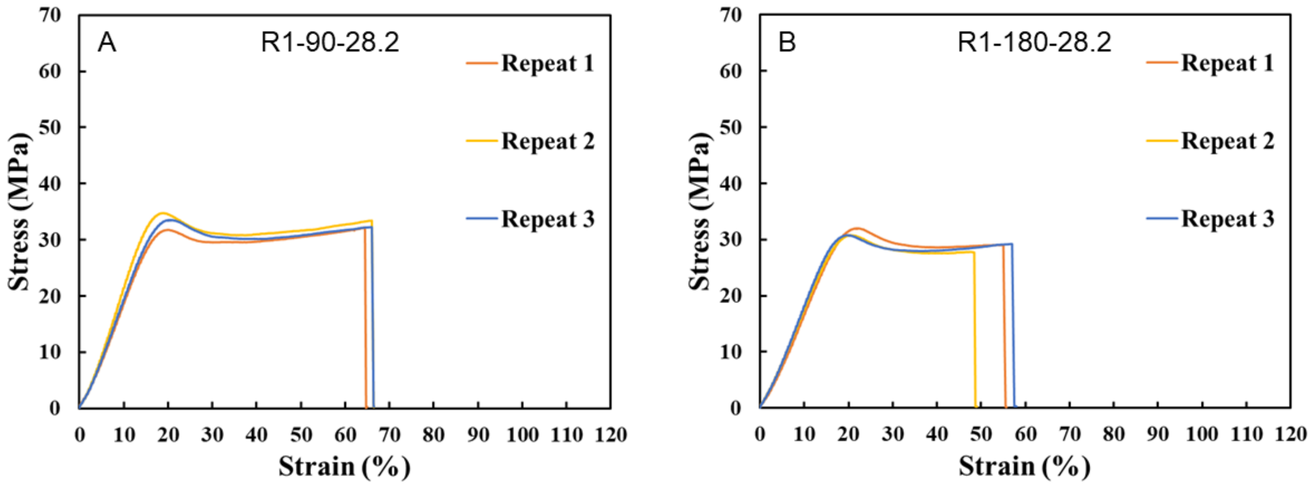

Figure S15: Stress-strain curves of objects 3D printed using R1-based resin A) R1-90-28.2 and B) R1-180-28.2.

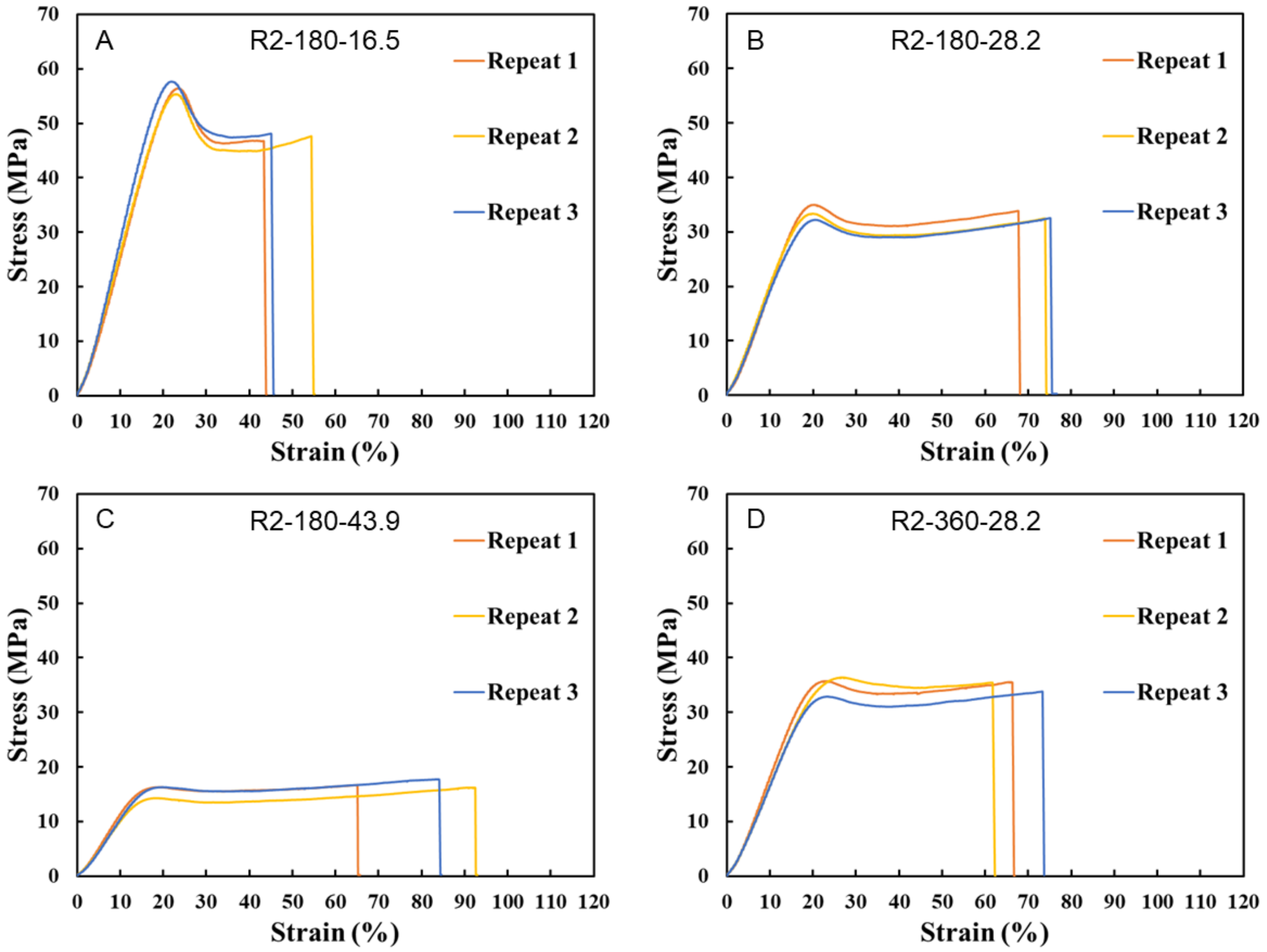

Figure S16: Stress-strain curves of objects 3D printed using R2-based resin A) R2-180-16.5, B) R2-180-28.2, C) R2-180-43.9 and D) R2-360-28.2.

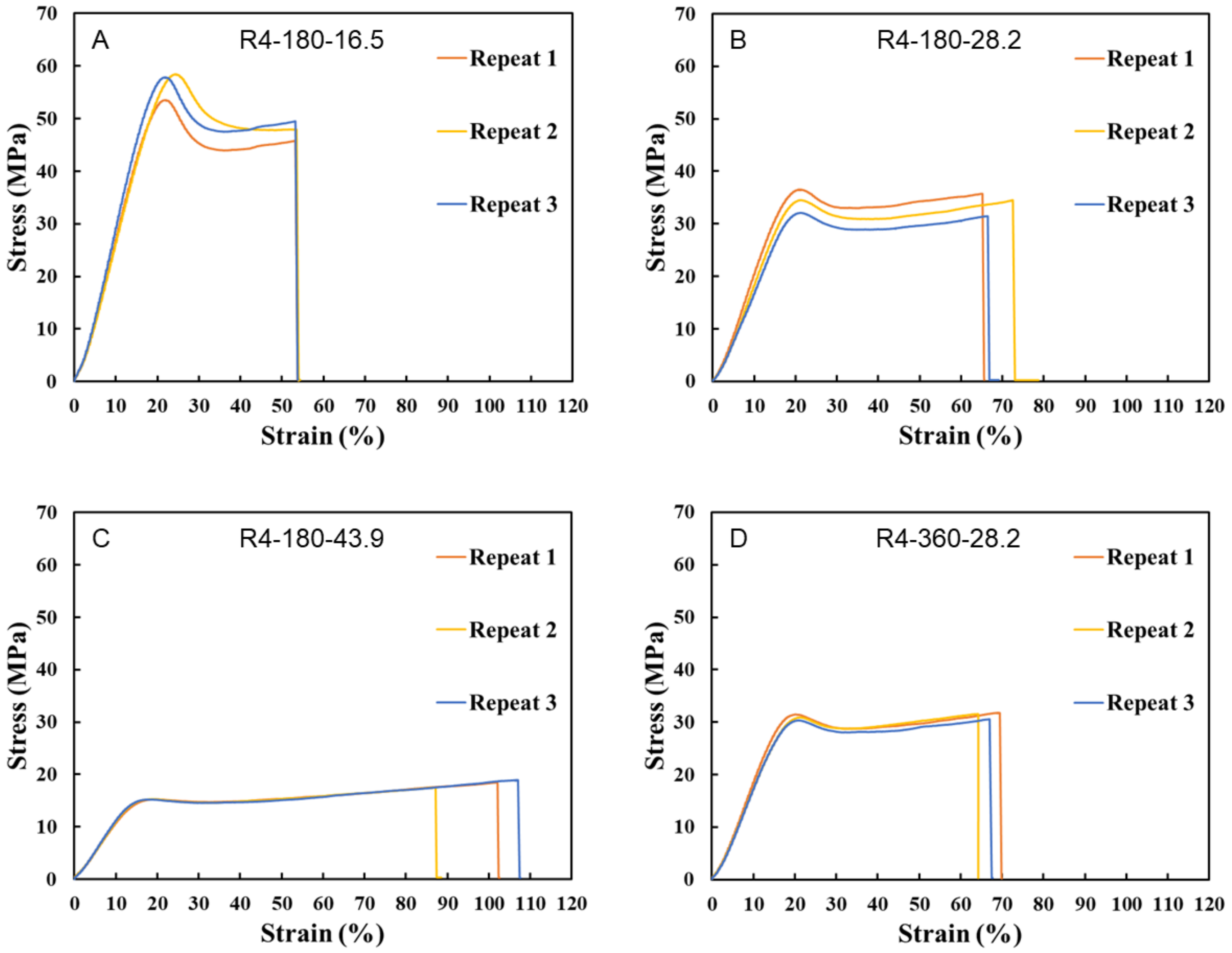

Figure S17: Stress-strain curves of objects 3D printed using R4-based resin A) R4-180-16.5, B) R4-180-28.2, C) R4-180-43.9 and D) R4-360-28.2.

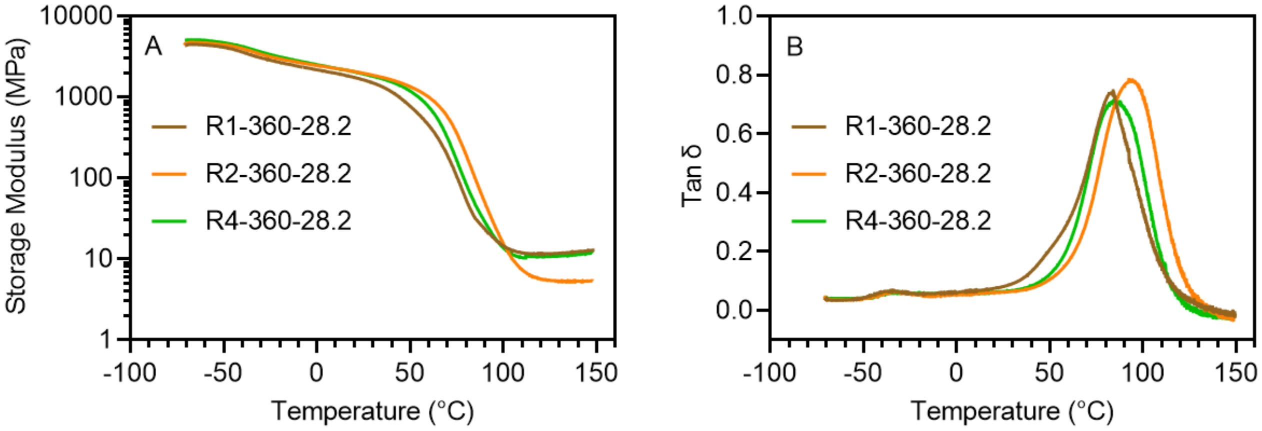

Figure S18: A) Storage modulus profile of materials 3D printed using resins containing 28.2wt% of macroCTAs (X_n = 360) with different architectures. B) tan δ profiles of materials 3D printed using resins containing 28.2wt% of macroCTAs (X_n = 360) with different architectures

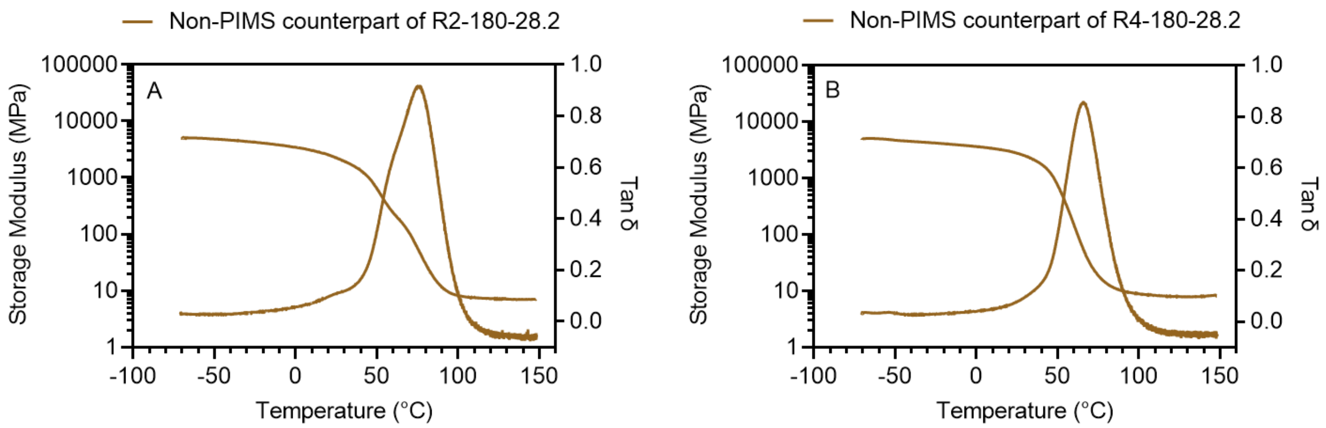

Figure S19: Storage modulus and tan δ profile of materials 3D printed using A) Non-PIMS resin counterpart of R2-180-28.2 and B) Non-PIMS resin counterpart of R4-180-28.2.

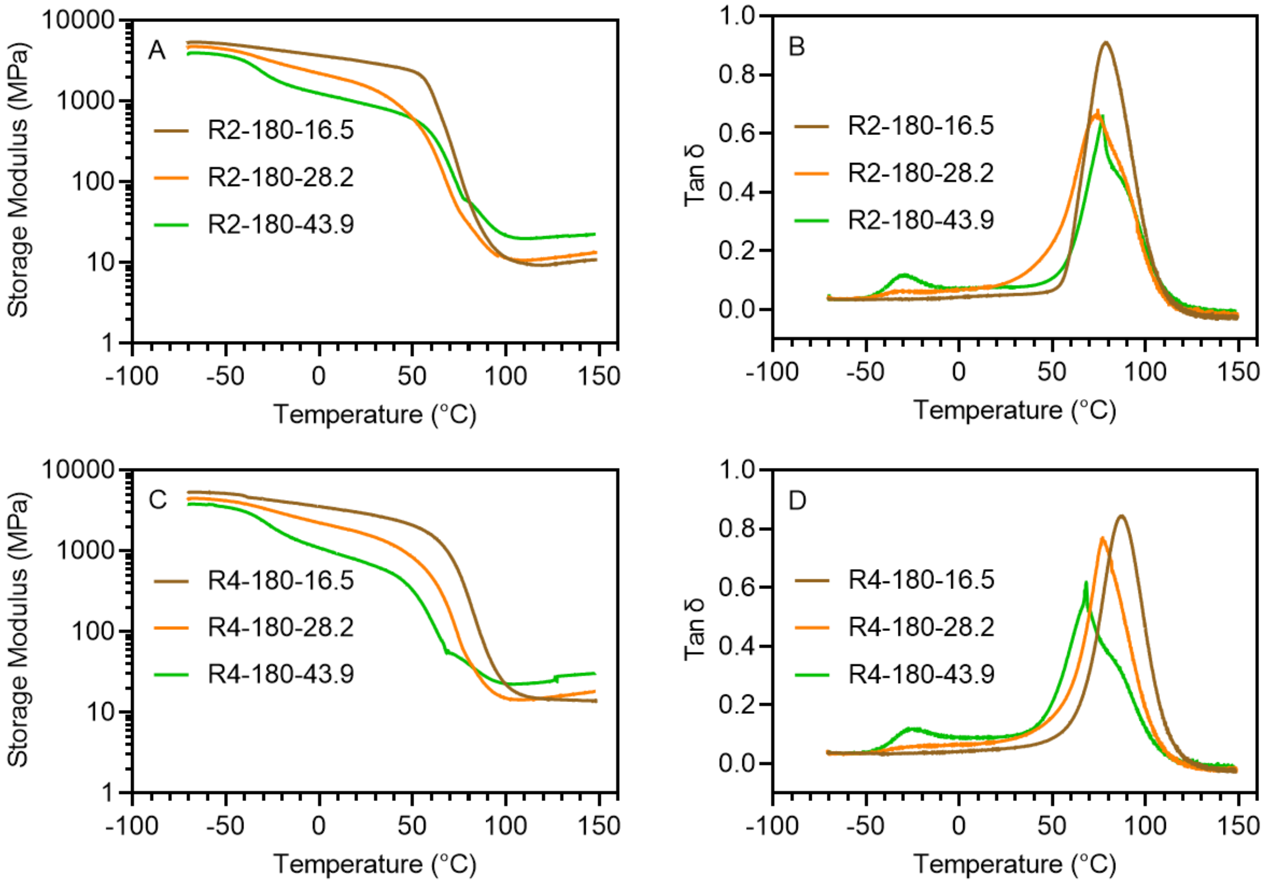

Figure S20: A) Storage modulus profile of materials 3D printed using resins containing different R2-180wt%. B) tan δ profiles of materials 3D printed using resins containing 28.2 wt% R2-180. C) Storage modulus profile of materials 3D printed using resins containing different R4-180 wt%. D) tan δ profiles of materials 3D printed using resins containing 28.2 wt% R4-180.

Table S3: Tensile properties of materials 3D printed with different resins.

	X_n	wt%	Tensile Strength (MPa)	Young's Modulus (MPa)	Elongation at break (%)
R1	90	28.2	33±1	222±19	66±1
	180	28.2	31±1	193±7	54±5
R2	180	16.2	57±1	305±23	48±6
	180	28.2	34±1	221±7	73±4
	180	43.9	16±1	119±7	80±12
	360	28.2	35±2	195±13	68±6
R4	180	16.2	57±3	324±18	54±1
	180	28.2	34±2	211±25	71±7
	180	43.9	15±0	116±3	100±10
	360	28.2	31±1	203±9	67±3

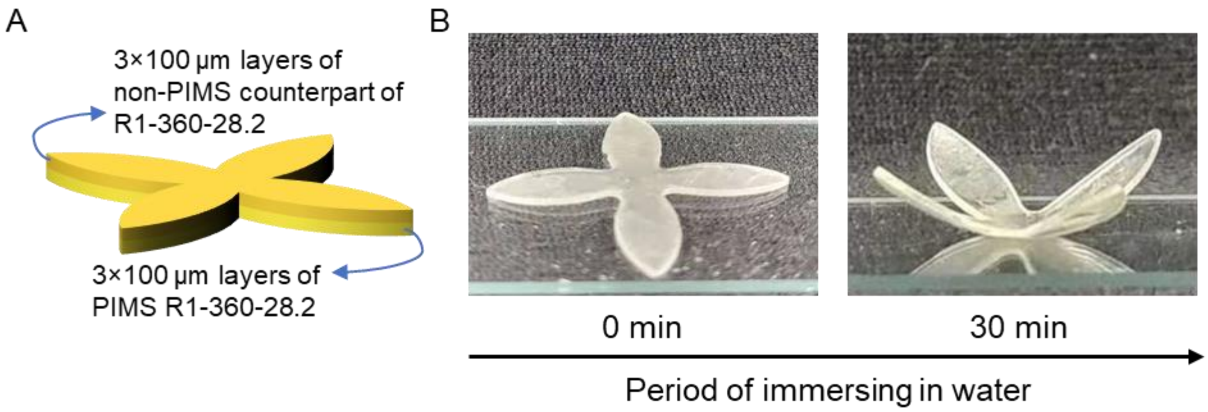

Figure S21: Solvent induced actuation using 3D printed PIMS materials. A) Model of the 3D printed flower. The top 3×100 μm layers of the flower were made using the non-PIMS counterpart of R1-360-28.2 while the bottom 3×100 μm layers of the flower were made using R1-360-28.2. B) Swelling induced actuation of a 3D printed flower in water.

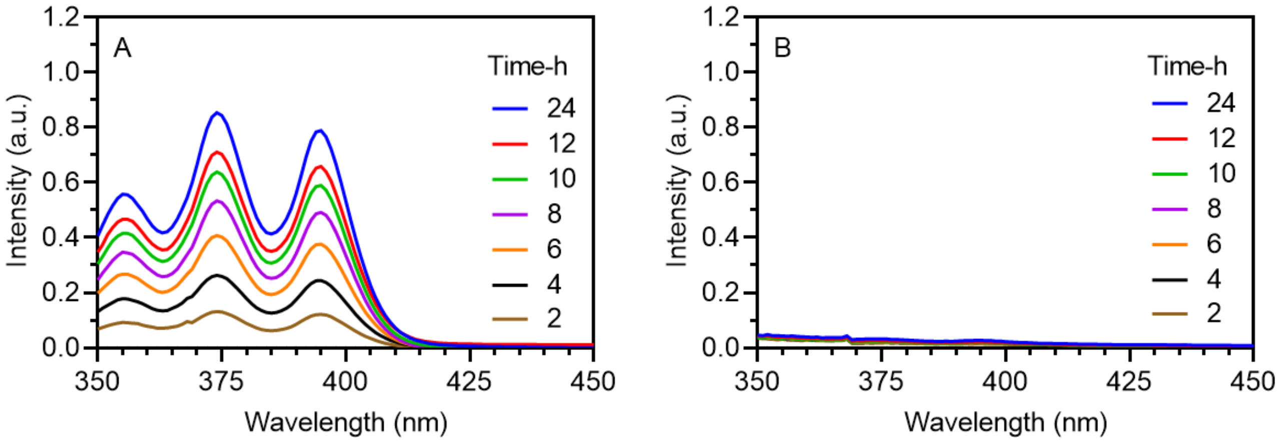

Figure S22: A) UV-vis. spectra of 9,10-diphenylanthracene in toluene released by a square prism 3D printed using A) R4-360-28.2 and B) Non-PIMS counterpart of R1-360-28.2.

Additional References:

[1] V. A. Bobrin, K. Lee, J. Zhang, N. Corrigan, C. Boyer, *Adv. Mater.* **2021**, 2107643.

[2] M. Teubner, R. Strey, *J. Chem. Phys.* **1987**, *87*, 3195-3200.

[3] A. Standard, *ASTM International: West Conshohocken, PA* **2014**.

[4] S. H. Chen, Chang, S. L., Strey, R. , *In Trends in Colloid and Interface Science IV; Zulauf, M., Lindner, P., Terech, P., Eds.; Dr. Dietrich Steinkopff Verlag* **1990**, *81*, 30-35.
